# Metabomatching: Using genetic association to identify metabolites in proton NMR spectroscopy

**DOI:** 10.1371/journal.pcbi.1005839

**Published:** 2017-12-01

**Authors:** Rico Rueedi, Roger Mallol, Johannes Raffler, David Lamparter, Nele Friedrich, Peter Vollenweider, Gérard Waeber, Gabi Kastenmüller, Zoltán Kutalik, Sven Bergmann

**Affiliations:** 1 Department of Computational Biology, University of Lausanne, Lausanne, Switzerland; 2 Swiss Institute of Bioinformatics, Lausanne, Switzerland; 3 Institute of Bioinformatics and Systems Biology, Helmholtz Zentrum München, German Research Center for Environmental Health, Neuherberg, Germany; 4 Institute of Clinical Chemistry and Laboratory Medicine, University Medicine Greifswald, Greifswald, Germany; 5 German Centre for Cardiovascular Research (DZHK), Partner site, Greifswald, Germany; 6 Department of Medicine, Internal Medicine, Lausanne University Hospital (CHUV), Lausanne, Switzerland; 7 German Center for Diabetes Research, Neuherberg, Germany; 8 Institute of Social and Preventive Medicine, Lausanne University Hospital (CHUV), Lausanne, Switzerland; 9 Department of Integrative Biomedical Sciences, University of Cape Town, Cape Town, South Africa; Centre for Research and Technology-Hellas, GREECE

## Abstract

A metabolome-wide genome-wide association study (mGWAS) aims to discover the effects of genetic variants on metabolome phenotypes. Most mGWASes use as phenotypes concentrations of limited sets of metabolites that can be identified and quantified from spectral information. In contrast, in an *untargeted* mGWAS both identification and quantification are forgone and, instead, all measured metabolome features are tested for association with genetic variants. While the untargeted approach does not discard data that may have eluded identification, the interpretation of associated features remains a challenge. To address this issue, we developed *metabomatching* to identify the metabolites underlying significant associations observed in untargeted mGWASes on proton NMR metabolome data. Metabomatching capitalizes on *genetic spiking*, the concept that because metabolome features associated with a genetic variant tend to correspond to the peaks of the NMR spectrum of the underlying metabolite, genetic association can allow for identification. Applied to the untargeted mGWASes in the SHIP and CoLaus cohorts and using 180 reference NMR spectra of the urine metabolome database, metabomatching successfully identified the underlying metabolite in 14 of 19, and 8 of 9 associations, respectively. The accuracy and efficiency of our method make it a strong contender for facilitating or complementing metabolomics analyses in large cohorts, where the availability of genetic, or other data, enables our approach, but targeted quantification is limited.

## Author summary

Metabolome-wide genome-wide association studies aim to discover how genetic variation affects metabolome traits. Such studies typically follow an acquire-identify-associate procedure: metabolome data are acquired experimentally, metabolites are identified in the experimental data and their concentrations quantified, and the metabolite concentrations are tested for association with genetic variants. The *untargeted* approach follows instead an acquire-associate-identify procedure: the experimental data are binned into *metabolome features*, and the features tested directly for genetic association. When the metabolome is measured by proton NMR spectroscopy, genetically associated features tend to correspond to peaks in the NMR spectrum of the underlying metabolites. This inherent property of the untargeted approach acts as a *genetic spiking* which informs on the identities of involved metabolites. *Metabomatching* is a method that uses genetic spiking information to identify the metabolite candidates, listed in a spectral database, most likely to underlie observed feature associations. Here, we present the method and its software, and evaluate its performance.

## Introduction

Since the seminal metabolome-wide genome-wide association study (mGWAS) by Gieger et al. in 2008 [1], mGWASes performed on blood and urine spectral metabolome phenotypes have uncovered an increasing part of the heritable variability of the human metabolome through the discovery of hundreds of genetically influenced metabolome phenotypes [2–4].

Most mGWASes use estimated metabolite concentrations as phenotypes [1, 5–10]. In such *targeted* mGWASes, metabolite concentrations are obtained by quantification [11] of spectral metabolome data produced by mass spectrometry (MS) or nuclear magnetic resonance (NMR) spectroscopy. While targeted approaches pave the way for reproducible metabolomics, only a fraction of the measured metabolome data is quantified into metabolite concentrations due to the arduous nature of metabolite identification [12–16]. In Rueedi et al. [17], we used an *untargeted approach* [18–20]: we binned then normalized the NMR data, and tested the resulting bin intensities, which we called *metabolome features*, for association with genotypes.

We then sought metabolite identification only for significantly associated metabolome features. To do so, we employed an inherent characteristic of the untargeted approach: *genetic spiking*. If the genetic component of a metabolite concentration is detected in the untargeted mGWAS, then the relevant genotype will associate with metabolome features that correspond to the peaks of the NMR spectrum of the metabolite. Much as metabolite spiking does by flooding a sample with a metabolite of interest, genetic spiking isolates, by genetic association, the spectrum of the genetically influenced metabolite. However, whereas the aim of metabolite spiking is to determine an unknown spectrum for a known metabolite, we developed *metabomatching* to use genetic spiking to identify an unknown metabolite from a known spectrum.

We previously showed that metabolite identification using the metabomatching procedure works in principle [17, 20], and applied it to identify the metabolite involved in a novel SNP-feature association. Here, we further develop metabomatching, present its core concepts and data, perform numerical simulations, and evaluate its performance on two sets of mGWAS data. We also present the metabomatching software, describe its implementation and settings, and highlight the best practices and pitfalls of its application.

## Materials and methods

Metabolome features are obtained by reducing the experimental NMR spectra into bins along the chemical shift range. This binning can be uniform or adaptive [13, 21–23], and is applied during standard processing of the NMR data, among other steps such as alignment or normalization. In an untargeted mGWAS, the quantification into metabolite concentrations is skipped, and the metabolome features are tested directly for association with genetic variants, such as single nucleotide polymorphisms (SNPs).

Any observed SNP-feature association, however, is but a proxy for the genetic effect of the SNP on the concentration of a certain metabolite. Metabolome features are derived from the NMR spectra of the measured samples, which in turn are combinations of the NMR spectra of the metabolites contained in the samples. Therefore, the genetic effect of a SNP on the concentration of a metabolite can be detected by the associations between the SNP and the metabolome features that match peaks in the NMR spectrum of the metabolite. This match between associated features and peaks of the spectrum allows, in principle, to identify the underlying metabolite.

**Fig 1:**
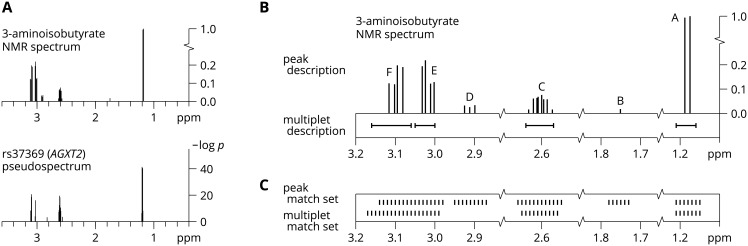
*AGXT2* pseudospectrum and 3-aminoisobutyrate NMR spectrum, descriptions, and metabomatching match sets. (A) The upper plot shows the experimental NMR spectrum of 3-aminoisobutyrate. The lower plot shows the (-log) *p*-values of the pseudospectrum of rs37369 in *AGXT2*, when *p* < 10^−3^. There is a close match between the experimental spectrum and the pseudospectrum, as the four sets of features that associate (*p* < 5 × 10^−8^) with rs37369 correspond to the principal peaks of the spectrum. (B) Taking a more detailed view of the spectrum descriptions (from HMDB), we see that the peaks of 3-aminoisobutyrate group into six clusters (labeled A through F). The multiplet ranges for clusters A, C, and E enclose their corresponding peaks well, padding by an average of 0.023 ppm. The multiplet range for cluster F is significantly wider, padding by 0.062 ppm. Approximating cluster areas as the product of the width of the cluster with the average height of the peaks in the cluster, then scaling, we find area-derived proton counts of 2.8, 0.7, 1.1, 1.2 for clusters A, C, E, and F, respectively. These counts are coherent with the listed proton counts of 3, 1, 1, and 1 for the respective multiplet ranges. Applying this same approximation for clusters B and D results in area-derived proton counts of 0.0, and 0.2. Because this implies corresponding multiplet proton counts of 0, we may consider the two spectrum descriptions essentially coherent, even though no multiplet ranges are listed for clusters B and D. (C) Match sets obtained from the peak and multiplet descriptions of the 3-aminoisobutyrate spectrum, for features resulting from a uniform NMR spectrum binning in 0.01 ppm increments, and with neighborhood parameter *δ* = 0.03 and 0.01, respectively. While the peak and multiplet descriptions of the 3-aminoisobutyrate NMR spectrum may be essentially coherent, their resulting match sets do differ, with 22 features unique to either one of the match sets.

To formalize the notion of genetic spiking, we call the collection of association *p*-values, effect sizes (*β*), and standard errors (*s*) resulting from the simple linear regressions between a SNP and all metabolome features the *pseudospectrum* of the SNP. As shown in Fig 1A for rs37369 in *AGXT2*, the pseudospectrum (−log) *p*-values mirror the NMR spectrum of the underlying metabolite 3-aminoisobutyrate almost exactly.

Metabomatching uses genetic spiking towards the identification of underlying metabolites: for a SNP that associates significantly with at least one metabolome feature, metabomatching compares the pseudospectrum of the SNP to the NMR spectrum of each metabolite listed in a supplied spectral database. It then scores and ranks the compared metabolites, such that high ranking metabolites are most likely to underlie the SNP-feature associations.

### Spectral databases

The default spectral database used by metabomatching is acquired from the Human Metabolome DataBase [24] (HMDB), which lists experimental proton NMR spectra for 835 metabolites. In HMDB, the spectrum of a metabolite is described in two ways: as a list of peaks, and as a list of multiplets (see Fig 1B). A peak is defined by a spectral position, expressed as a chemical shift in parts per million (ppm), and a relative NMR intensity, that is the peak height expressed relative to the highest peak in the spectrum. A multiplet is defined by a chemical shift range, and a proton count. Peaks group into clusters, and for each such cluster in the peak description, there is, generally, a corresponding multiplet in the multiplet description whose range encloses the cluster. Furthermore, the area under the curve delimiting the peaks of a cluster can be related to the proton count of the corresponding multiplet [25]. The two descriptions are usually, but not always, coherent.

Alternatively, metabomatching can use a database acquired from the Biological Magnetic Resonance dataBank [26] (BMRB), which lists experimental proton NMR spectra for 670 metabolites. In BMRB, the spectrum of a metabolite is described only as a list of peaks. Each metabolite, however, may have several peak description spectra, obtained in different experiments.

Both HMDB and BMRB collect information on any metabolites found in the human body. As a result, many of the spectra contained in the full spectral databases may be irrelevant for a specific mGWAS, typically because the corresponding metabolites may not be contained in the studied biofluid. Metabomatching can therefore also use specific subsets of the full spectral databases. For urine, the spectral database is derived from the urine metabolome database (UMDB) [27] and contains proton NMR spectra for 180 metabolites, 124 if based on BMRB. For serum, the spectral database is derived from the work of Gowda et al. [12] and contains proton NMR spectra for 67 metabolites if based on HMDB, 49 if based on BMRB.

### Standard method

For the comparison of pseudospectra to reference spectra, we introduce a feature match set *F*_*δ*_(*m*) for every metabolite *m* in the reference database. *F*_*δ*_(*m*) is defined to contain all features *f* within a neighborhood of *δ* ppm of any spectrum peak listed in the peak description of *m* (see Fig 1C). For the pseudospectrum of a given SNP *r* and the spectrum of every metabolite *m*, we compute the match sum

$$\begin{array}{c} \sum_{f\in F_{\delta }\left(m\right)}\frac{\beta_{rf}^{2}}{s_{rf}^{2}}, \end{array}$$ (1)

with *β*_*rf*_ the effect size and *s*_*rf*_ the standard error of the association between SNP *r* and feature *f*. Even though the features are usually not independent, we consider the match sum to be *χ*^2^-distributed with |*F*_*δ*_(*m*)| degrees of freedom, so as to define the score for the tested metabolite as the negative logarithm of the corresponding *p*-value. As a result, while we use the scores to rank metabolites for a given SNP, the scores do not inform on the statistical significance of a spectrum-pseudospectrum match, nor do we compare scores obtained for the pseudospectra of different SNPs.

### Settings

Because multiplet descriptions of the reference NMR spectra in HMDB can significantly differ from peak descriptions, they can be considered as composing a separate spectral database. To use this set, or its corresponding biofluid specific subsets, metabomatching can be run in *multiplet mode*, instead of the standard *peak mode* described above. The match set *F*_*δ*_(*m*) used to compute the match sum (1) for *m* is then defined to contain all features *f* falling in, or within *δ* of, any multiplet range of metabolite *m* (see Fig 1C). Because multiplet ranges tend to pad their corresponding peak cluster, the neighborhood parameter *δ* takes a smaller value in multiplet mode than in peak mode. The resulting match sets are then comparable to their peak mode counterparts, in general. However, differences between the two descriptions, in cluster position, size, or even presence, occur for about 10% of metabolites in HMDB. These differences can significantly affect metabomatching results.

Metabolome features that are common to the spectrum of a metabolite present in the study samples correlate, and metabomatching can be set to take this correlation into account. The correlation is strongest among neighboring features, which may be common to multiple metabolites of spectra containing similar peak clusters, but also appears in features corresponding to peaks in different spectrum clusters. Heuristically however, only the correlation between neighboring features is detrimental to metabomatching, and decorrelation is therefore applied only to feature neighborhoods. Given the user-provided feature-feature correlation matrix $\hat{C}$, match sum (1) is then modified to

$$\begin{array}{c} \sum_{f,g\in F_{\delta }\left(m\right)}\frac{\beta_{rf}}{s_{rf}}C_{\delta ;fg}^{-1}\frac{\beta_{rg}}{s_{rg}}, \end{array}$$ (2)

where $C_{\delta ;fg}≐\left(1-\lambda \right){\hat{C}}_{fg}J_{\delta ;fg}+\lambda I_{fg}$ provides decorrelation, with λ ∈ [0, 1] the shrinkage parameter [28], which serves to regularize. Restriction to feature neighborhoods results from the block diagonal matrix *J*_*δ*_, with *J*_*δ*;*fg*_ = 1 if *f* and *g* are members of the same neighborhood, that is if they are connected by a sequence of features in *F*_*δ*_(*m*) each at most 2*δ* ppm apart, and *I* the identity matrix.

Metabomatching includes two variants for cases where a SNP affects a pair of metabolites: *2-compound* metabomatching if the effects are of equal directions, and ±-metabomatching if the effects are of opposite directions. For 2-compound metabomatching, we compute the match sum for pairs of metabolites by running the sum over pair match sets, defined as *F*_*δ*_(*m*_1_, *m*_2_) ≐ *F*_*δ*_(*m*_1_) ∪ *F*_*δ*_(*m*_2_). Metabolite pairs are accordingly scored and ranked. In ±-metabomatching, standard (*1-compound*) metabomatching is run separately for each effect direction, setting to 0 the effect size for associations in the other direction that exceed a user-provided *p*-value threshold. 2-compound and ±-metabomatching can be combined into *±-2-compound* metabomatching for SNPs affecting at most one pair of metabolites in each direction.

By squaring *β*/*s* in match sum (1) or (2), *χ*^2^-scoring increases signal to noise ratio, both by amplifying the contribution of strongly associated features to metabomatching scores, and by ignoring effect directions. This increase applies indiscriminately, however, and may actually favor competing metabolites more than the metabolite to identify. Therefore, for pseudospectra where this increase is not necessary, such as those produced in mGWASes of high statistical power, for example, stronger matches may be obtained with *Z*-scoring. Here, scores are computed according to the match sum

$$\begin{array}{c} \sum_{f\in F_{\delta }\left(m\right)}\frac{\beta_{rf}}{s_{rf}} \end{array}$$ (3)

which we consider to be normally distributed, under the null hypothesis, with zero mean and variance |*F*_*δ*_(*m*)|, even though the sampled features are not independent. To apply decorrelation in *Z*-scoring metabomatching, match sum (3) is not modified, but the variance is computed as |∑_*fg*_ *C*_*δ*;*fg*_|, with *C*_*δ*_ the block diagonal matrix as previously defined. As in *χ*^2^-scoring, multiplet-mode and 2-compound variants applied by using the corresponding match sets in match sum (3). Because *Z*-scoring is explicitly sensitive to effect directions, ±-metabomatching is not required for SNPs affecting two metabolites with opposite effect directions. However, the separate presentation of results of ±-metabomatching may be useful in cases where the effect sizes are such as to cause metabolites matched with one effect direction to systematically outrank metabolites matched with the other direction.

### Output

**Fig 2:**
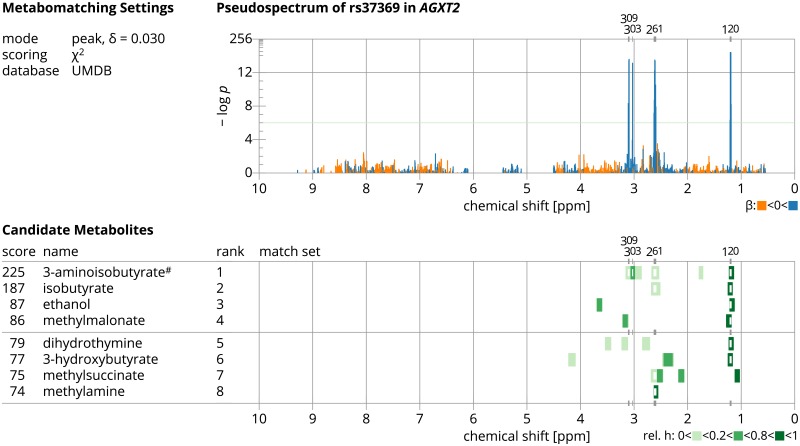
Metabomatching results figure. The metabomatching results figure, shown here for the same *AGXT2* pseudospectrum as in Fig 1. The figure shows the metabomatching settings used, the pseudospectrum with features color-coded by effect size, and, for the eight highest ranked candidate metabolites, the score, name, and reference NMR spectrum with match sets color-coded according to the height of the NMR spectrum peak they derive from.

To summarize, metabomatching is run for a given pseudospectrum: against a set of match sets, defined by the selected spectral reference database, the mode, and neighborhood parameter *δ*; where appropriate, as 1-compound, 2-compound, ±-, or ±-2-compound variant; and depending on performance, with or without decorrelation, and with *χ*^2^- or *Z*-scoring. Metabomatching outputs the score for each metabolite in the spectral database, and produces a figure showing the pseudospectrum and the spectra of the highest ranked candidate metabolites (Fig 2).

### Simulation

We bin the chemical shift range [0, 10] uniformly, in 0.01 ppm increments, and round reference spectra to the binning. We express the spectrum of each metabolite as a vector ***h***^*m*^, with $h_{j}^{m}$ the height of the peak in bin *j*, set to 0 if the spectrum does not include bin *j*, and define the *size* of the spectrum as $s^{m}≐\sum_{j}h_{j}^{m}$. To model the genetic association between a SNP and metabolite *m*, we randomly assign a genotype *g*_*i*_ ∈ {0, 1, 2} to each individual (*i* ∈ [1, 400]), according to a minor allele frequency of 0.2, and build the feature metabolome ***M***^0^ of elements

$$\begin{array}{c} M_{ij}^{0}≐\beta h_{j}^{m}g_{i}+N\left(0,1\right). \end{array}$$ (4)

Because the number of individuals, the minor allele frequency and the amplitude of noise are fixed, the strength of the association is controlled fully by the choice of effect size *β*. We then associate the metabolome ***M***^0^ with the genotype ***g***, and apply metabomatching to the resulting pseudospectrum. For each metabolite, we repeat this procedure 1 000 times, and compute $r_{90}^{m}$, the 90th percentile over the 1 000 ranks of *m*. We consider metabomatching successful for *m* if $r_{90}^{m}=1$.

**Fig 3:**
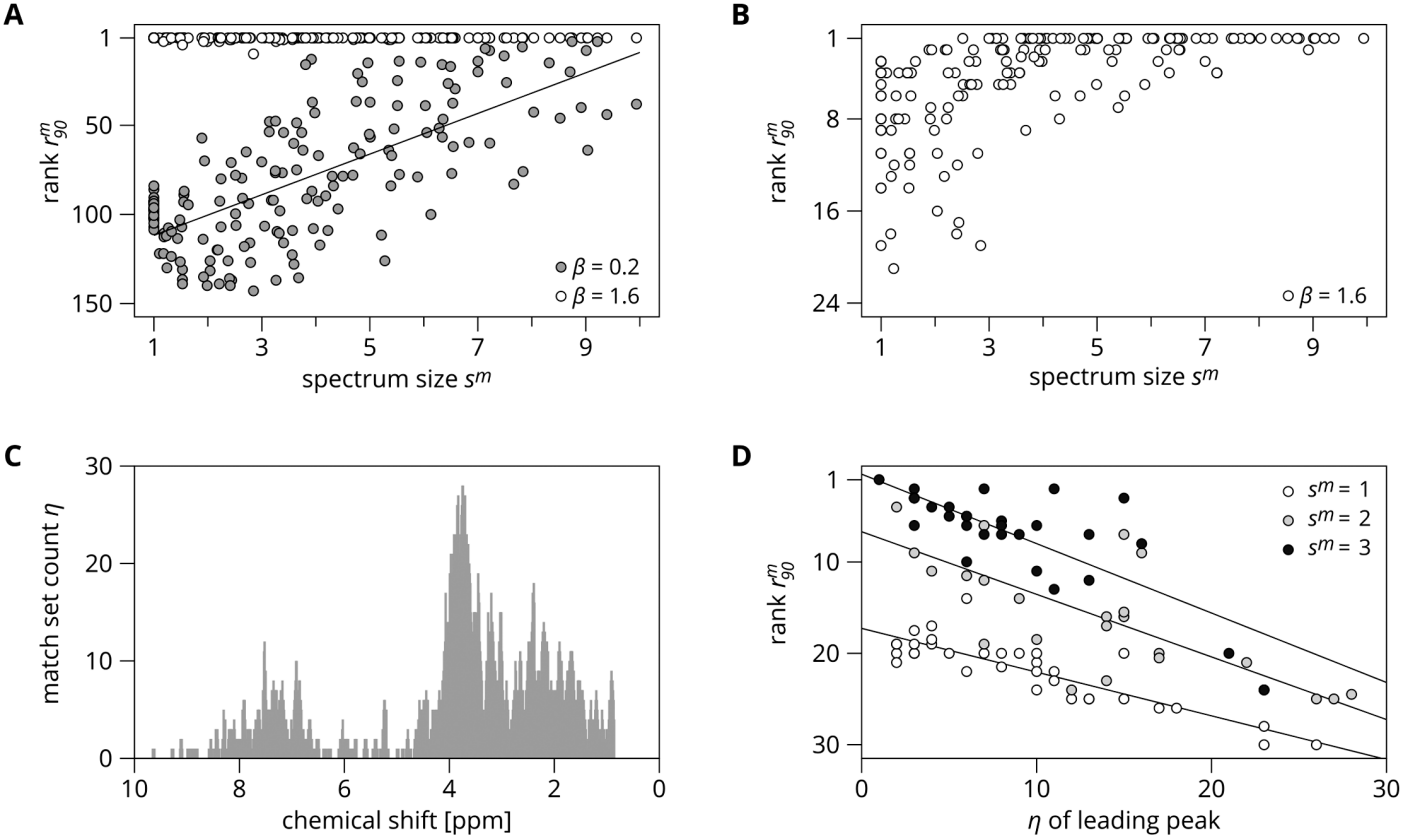
Metabomatching results on simulated metabolomes. **A.** Metabomatching performance, measured as $r_{90}^{m}$, the 90^th^ percentile of 1 000 ranks obtained for *m* by metabomatching pseudospectra build from the association with ***M***^0^, for *β* = 0.2 (filled dots) and *β* = 1.6 (empty dots), as a function of the size of the metabolite spectrum.For *β* = 0.2, the correlation between $r_{90}^{m}$ and *s*^*m*^ is −0.71, with *p* ∼ 10^−26^. **B.** As in (**A**), but for metabolome ***M***^*α*^, with *β* = 1.6, *N*_*a*_ = 64 and *α* = 0.6. **C.** For UMDB and *δ* = 0.02, number of match sets (*η*) that contain each feature (uniform binning in 0.01 ppm increments). **D.** Metabomatching performance, measured as $r_{90}^{m}$, for ***M***^*α*^, with *β* = 1.6, *N*_*a*_ = 64 and *α* = 0.6, as a function of *η* of the leading peak of the metabolite spectrum, that is the peak with *h*_*j*_ = 1. Metabolite sizes are rounded. For *s*^*m*^ = 1, 2, and 3, respectively, correlations *ρ* are 0.86, 0.79, and 0.76, with *p*-values ∼10^−10^, 10^−4^, and 10^−4^.

From the results of this simple model, shown in Fig 3A for UMDB, we can make two important observations. First, that if the effect size is large enough, metabomatching can identify any metabolite. Second, that the performance of metabomatching, characterized here by $r_{90}^{m}$, is strongly correlated with the spectrum size.

We then add genetic noise to the model, in the form of *N*_*a*_ randomly drawn features that also associate, with a randomly drawn direction, with genotype ***g***. We insert these genetic noise features in the model by adding the terms *a*_*j*_ ∈ {−1, 0, 1}, such that ∑_*j*_ |*a*_*j*_| = *N*_*a*_, when building the feature metabolome ***M***^*α*^ of elements

$$\begin{array}{c} M_{ij}^{\alpha }≐\beta h_{j}^{m}g_{i}+\alpha \beta g_{i}a_{j}+N\left(0,1\right), \end{array}$$

where *α* < 1. As the amount *N*_*a*_, or amplitude *α*, of genetic noise increases, metabolite *m* faces wider, respectively stronger, competition from other metabolites in the spectral database.

When *β* is small, random noise still determines metabomatching performance, and $r_{90}^{m}$ is similar to that for metabolome ***M***^0^ shown in Fig 3A. When *β* is large, however, genetic noise dominates. As shown in Fig 3B (and S4 Fig for other settings and for both UMDB and HMDB), metabomatching can then no longer identify all metabolites consistently, because other metabolites in the database outscore *m* by matching genetic noise features. Some of these other metabolites may obtain their score from genetic noise features *only*, but true competition for *m* is provided by metabolites that match both genetic noise features and features of *m*. Because these competing metabolites have spectra similar to the spectrum of *m*, they tend to be viable metabomatching candidates. For metabolites with a single peak *f*, we can count the number of metabolites of match set that contain *f* to determine the size of this competing group. In Fig 3C, we show this number, *η*(*f*) for UMDB, and in Fig 3D we see that $r_{90}^{m}$ for metabolites of size 1 correlates strongly with *η*. For larger spectra, where we take *η* for the lead feature (the one of height 1), the correlation holds, but *η* is less representative of the size of the competing group.

## Results

We first tested metabomatching on pseudospectra obtained in the urine NMR mGWAS [17] in the CoLaus study [29]. NMR data were aligned, normalized, and uniformly binned in 0.005 ppm increments. The resulting untargeted metabolome contained 1,276 features for 835 individuals. As references, we used SNP-metabolite associations that were previously reported in targeted mGWASes on urine NMR [8, 18, 20] with a *p*-value below 10^−5^ and involving a metabolite for which an NMR spectrum is listed in UMDB. If a CoLaus SNP located within 500kb of the reference SNP associated with *p* < 10^−6^ with at least one feature contained in the match set of the reference metabolite (with *δ* = 0.03 in peak mode, 0.01 in multiplet mode), we considered the CoLaus SNP pseudospectrum *testable*, and assumed the reference metabolite to be the metabolite underlying the SNP-feature association. This resulted in nine testable pseudospectra, each with a single reference metabolite.

Metabomatching with default settings (peak mode, *χ*^2^-scoring, and without decorrelation), and using the urine specific UMDB reference database, was successful for eight of the nine testable pseudospectra, ranking the reference metabolite first three times and in the top ten five times (column ${\text{P}}_{\text{C}}^{\text{X}}$ of Table 1, detailed results in S1 Fig). For the *SOSTDC1* SNP, the pseudospectrum (S1C Fig) shows strong inflation across almost the entire chemical shift range, making metabomatching fail systematically. Metabomatching in multiplet mode performed better overall (column ${\text{M}}_{\text{C}}^{\text{X}}$), ranking the reference metabolites first six times and second twice, though the performance was qualitatively different only for the *HPD* SNP pseudospectrum, for which the testable association involved a different reference metabolite (S1F and S1G Fig). Decorrelation had little effect on rankings, in either mode, provided a shrinkage parameter λ greater than 0.1 was used (results for λ = 0.5 in Table 1 columns ${\text{P}}_{\text{D}}^{\text{X}}$ and ${\text{M}}_{\text{D}}^{\text{X}}$, other values of λ in S1 Table).

**Table 1:** CoLaus metabomatching results. Ranks of reference metabolites obtained for CoLaus pseudospectra, with UMDB as the spectral reference database, and with: peak- (P) or multiplet-mode (M), *χ*^2^- (X) or *Z*-scoring (Z), and without (C) or with decorrelation (D). Neighborhood parameter is *δ* = 0.03 in peak-mode, 0.01 in multiplet-mode. Shrinkage parameter is λ = 0.5 for decorrelation, 1 without. Reference metabolites are obtained from testable associations collected from targeted mGWAS [8, 18, 19]. Squares (□) indicate ranks not in the top 10% of UMDB listed metabolites, that is ranks greater than 18. Individual metabomatching figures including the eight highest ranked metabolite candidates for each pseudospectrum can be found in S1 Fig. Due to the differences in the peak and multiplet descriptions, the association of the *HPD* SNP with *α*-hydroxyisobutyrate is testable only in peak mode (S1F Fig), the association with 3-hydroxyisovalerate only in multiplet mode (S1G Fig).

Locus		Reference Association			Feature Association			Ranks							
Gene	Chr	SNP	Metabolite	*p*	SNP	Feat.	*p*	${\text{P}}_{\text{C}}^{\text{X}}$	${\text{P}}_{\text{D}}^{\text{X}}$	${\text{M}}_{\text{C}}^{\text{X}}$	${\text{M}}_{\text{D}}^{\text{X}}$	${\text{P}}_{\text{C}}^{\text{Z}}$	${\text{P}}_{\text{D}}^{\text{Z}}$	${\text{M}}_{\text{C}}^{\text{Z}}$	${\text{M}}_{\text{D}}^{\text{Z}}$
*SLC6A20*	3	rs17279437	dimethylglycine	1.1 × 10^−46^	rs4327428	2.933	7.3 × 10^−10^	2	1	2	1	□	7	12	1
*AGXT2*	5	rs37369	3-aminoisobutyrate	2.4 × 10^−252^	rs37369	1.203	3.9 × 10^−42^	1	1	1	1	2	1	1	1
*SOSTDC1*	7	rs10238442	taurine	4.0 × 10^−6^	rs17169536	3.393	7.6 × 10^−7^	□	□	□	12	□	□	□	□
*PYROXD2*	10	rs4539242	trimethylamine	2.8 × 10^−23^	rs4488133	2.857	1.3 × 10^−98^	5	4	2	2	4	1	4	3
*SLC6A13*	12	rs11613331	3-aminoisobutyrate	2.5 × 10^−15^	rs10774021	1.193	9.5 × 10^−10^	4	4	1	1	14	5	□	□
*HPD*	12	rs4760099	*α*-hydroxyisobutyrate	2.2 × 10^−80^	rs7314056	1.363	9.8 × 10^−11^	3	1			□	□		
			3-hydroxyisovalerate	2.4 × 10^−7^						2	5			□	□
*PNMT*	17	rs8069451	tyrosine	7.9 × 10^−22^	rs676882	6.897	1.8 × 10^−8^	3	3	1	1	□	□	□	□
*SLC7A9*	19	rs8101881	lysine	3.3 × 10^−25^	rs6510300	1.733	3.9 × 10^−15^	1	1	1	1	1	2	1	2
*UPS9*	19	rs13343495	sucrose	3.4 × 10^−6^	rs17273533	5.417	4.0 × 10^−7^	1	2	1	1	2	2	3	1

*Z*-scoring metabomatching properly ranked the reference metabolites for the pseudospectra characterized by the strongest associations, that is those for SNPs in *AGXT2*, *PYROXD2*, and *SLC7A9*. Pseudospectra with weaker associations fared worse, with *Z*-scoring metabomatching ranks significantly lower than their *χ*^2^-scoring counterparts, except for the *UPS9* pseudospectrum. For the *SLC6A20* and *SLC6A13* pseudospectra, the reference metabolite is outranked by a number of metabolites of spectra that obtain their score by matching a group of strongly correlated features. Applying decorrelation reduces this correlation-based score, thereby significantly improving the rank of the reference metabolite in both peak- and multiplet-mode (see S3 Fig).

Using the full HMDB spectral database (S2 Table), metabomatching ranked the reference metabolites for *PYROXD2*, *PNMT*, *HPD* markedly lower, due to stronger competition among the larger pool of candidate metabolites. Using the UMRB or BMRB spectral databases (S2 Table), metabomatching ranks the reference metabolite for *PNMT* lower, for *PYROXD2* higher, but is otherwise comparable to UMDB or HMDB, respectively.

We then tested metabomatching on pseudospectra obtained in the urine mGWAS [20] in the SHIP study [30]. NMR data were normalized, binned in 0.0005 ppm increments, then processed with FOCUS [31]. The resulting untargeted metabolome contained 166 features for 3,861 individuals. In addition, NMR data were manually annotated using Chenomx NMR Suite 7.0. The resulting targeted metabolome contained the concentrations of 59 metabolites for the same 3,861 individuals. Having both metabolome features and metabolite concentrations in the same sample allowed for the direct comparison of SNP-metabolite association results via metabomatching with targeted metabolite quantification followed by association. We considered the pseudospectrum of a SNP associating with *p* < 10^−6^ with both a metabolite and at least one feature contained in the metabolite spectrum testable. This resulted in nineteen testable SNP-metabolite associations involving fourteen SNPs.

Because testing is in the same samples, and because of the higher sample size of the study, metabomatching results for SHIP pseudospectra are more nuanced than they were for CoLaus pseudospectra. For the nine SNPs that associate with a single metabolite, metabomatching in default settings ranked the reference metabolite first five times, and in the top ten four times (see Table 2 column ${\text{P}}_{\text{C}}^{\text{X}}$, detailed results in S2 Fig). For the *CPS1* and *HPD* SNPs, which associate with two metabolites each, metabomatching ranked one metabolite first, the second in the top ten, and 2-compound metabomatching ranked the reference metabolite pair first. The pseudospectra for the three remaining SNPs are more complex. While the *NAT2* SNP only associates with formate, its pseudospectrum (S2N Fig) indicates the presence of additional associations, in both effect directions. We therefore applied ±-2-compound metabomatching (S2O Fig), which ranks a metabolite pair that includes formate first, in the *β* > 0 direction. With associations with three reference metabolites, the *PNMT* SNP pseudosepctrum (S2U Fig) is too complex for metabomatching, or 2-compound metabomatching, to provide any of the reference metabolites as plausible candidates. The *SLC6A19* SNP pseudospectrum (S2J Fig) is similar to the *PNMT* SNP pseudospectrum, but with weaker associations. Because the secondary associations are closer to the noise background, metabomatching still provides top ten ranks for the two reference metabolites. 2-compound metabomatching, however, does not properly rank the reference pair.

**Table 2:** SHIP metabomatching results. Metabomatching ranks of reference metabolites obtained for SHIP pseudospectra, with UMDB as the spectral reference database, and with: peak- (P) or multiplet-mode (M), *χ*^2^- (X) or *Z*-scoring (Z), and without (C) or with decorrelation (D). Neighborhood parameter is *δ* = 0.03 in peak-mode and 0.01 in multiplet-mode. Shrinkage parameter is λ = 0.5 for decorrelation, 1 without. Ranks obtained with 2-compound metabomatching are shown in bold, those obtained with ±-2-compound metabomatching in bold and italic. Squares (□) indicate ranks not in the top 10% of UMDB listed metabolites, that is ranks greater than 18. Individual metabomatching figures including the eight highest ranked metabolite candidates for each pseudospectrum can be found in S2 Fig.

Locus			Reference Association		Feature Association		Ranks							
Gene	Chr	SNP	Metabolite	*p*	Feature	*p*	${\text{P}}_{\text{C}}^{\text{X}}$	${\text{P}}_{\text{D}}^{\text{X}}$	${\text{P}}_{\text{C}}^{\text{Z}}$	${\text{P}}_{\text{D}}^{\text{Z}}$	${\text{M}}_{\text{C}}^{\text{X}}$	${\text{M}}_{\text{D}}^{\text{X}}$	${\text{M}}_{\text{C}}^{\text{Z}}$	${\text{M}}_{\text{D}}^{\text{Z}}$
*DAB1*	1	rs558475	hippurate	3.9 × 10^−7^	3.949	3.6 × 10^−8^	1	1	1	1	1	1	1	1
*CPS1*	2	rs2216405	glycine	2.9 × 10^−11^	3.555	4.9 × 10^−9^	**1**	**1**	□	□	3	1	□	□
			creatine	7.5 × 10^−11^			**1**	**1**	□	□	16	11	□	□
*XYLB*	3	rs2070486	glycolate	1.4 × 10^−9^	3.937	2.4 × 10^−9^	2	1	5	2	16	16	□	□
*SLC6A20*	3	rs17279437	dimethylglycine	1.1 × 10^−46^	2.916	1.1 × 10^−21^	1	2	2	2	1	1	1	1
*ENTPPL*	4	rs7654111	ethanolamine	2.3 × 10^−26^	3.126	5.0 × 10^−16^	1	1	4	5	1	1	2	2
*SLC6A19*	5	rs7719875	histidine	2.4 × 10^−14^	6.877	6.4 × 10^−12^	8	8	10	11	10	8	8	8
			tyrosine	6.5 × 10^−10^			9	13	12	12	12	14	□	14
*AGXT2*	5	rs37369	3-aminoisobutyrate	2.4 × 10^−252^	1.171	3.7 × 10^−252^	1	1	2	2	1	1	1	1
*DMGDH*	5	rs248386	dimethylglycine	1.0 × 10^−13^	2.916	1.8 × 10^−8^	5	5	□	□	2	1	□	□
*SLC36A2*	5	rs3846710	glycine	1.1 × 10^−10^	3.555	6.3 × 10^−9^	1	1	2	2	1	1	2	5
*NAT2*	8	rs1495743	formate	9.5 × 10^−60^	3.189	1.6 × 10^−104^	***1***	***1***	**1**	**1**	***1***	***1***	**1**	**1**
*SLC6A13*	12	rs11613331	3-aminoisobutyrate	2.5 × 10^−15^	1.190	5.0 × 10^−16^	5	5	3	3	4	4	3	3
*HPD*	12	rs4760099	*α*-hydroxyisobutyrate	2.2 × 10^−80^	1.345	2.2 × 10^−64^	**1**	**1**	**1**	**1**	2	1	1	1
			3-hydroxyisovalerate	2.4 × 10^−7^			**1**	**1**	**1**	**1**	3	4	2	5
*PNMT*	17	rs8069451	tyrosine	7.9 × 10^−22^	6.877	4.4 × 10^−17^	□	□	□	□	□	□	□	□
			histidine	7.3 × 10^−21^			□	□	□	□	□	□	□	□
			alanine	2.3 × 10^−11^			18	18	16	□	17	□	15	□
*SCL7A9*	19	rs8112297	lysine	5.0 × 10^−16^	3.003	9.4 × 10^−7^	9	9	15	17	15	11	17	□

Metabomatching in multiplet mode shows similar results for most SNPs (column ${\text{M}}_{\text{C}}^{\text{X}}$). However, for the *CPS1*, *XYLB*, *HPD* SNPs, the multiplet ranges describing the spectra of the respective reference metabolites are wide (between 0.16 and 0.28 ppm) even though each range encloses only a single peak. The resulting multiplet-mode neighborhoods have a higher number of degrees of freedom than their peak-mode counterparts, yet produce similar sum values. This lowers the scores of the reference metabolites, which are then outranked by competing metabolites, particularly in 2-compound metabomatching (S2D, S2E, S2G, S2S and S2T Fig).

*Z*-scoring metabomatching underperforms *χ*^2^-scoring overall (columns ${\text{P}}_{\text{C}}^{\text{Z}}$ and ${\text{M}}_{\text{C}}^{\text{Z}}$), yet *Z*-scoring ranks obtained for the reference metabolites are close to their corresponding *χ*^2^-scoring ranks for all but two pseudospectra. For the *CPS1* and *DMGDH* pseudospectra, the association of the lead feature is too weak to compensate for the associations of opposite effect direction of other features captured by the match sets of the corresponding reference metabolites (see S2B and S2L Fig). The resulting penalties incurred under *Z*-scoring produce low reference metabolite ranks.

FOCUS combines neighboring features into a single representative feature, obtained either by peak picking or by integration of the NMR curve in the neighborhood. As a result, the effect on metabomatching ranks of correlation in feature neighborhoods is weaker because neighborhoods contain fewer features after FOCUS processing. Correspondingly, ranks with decorrelation are essentially equal to ranks without decorrelation (columns ${\text{P}}_{\text{D}}^{\text{X}}$, ${\text{P}}_{\text{D}}^{\text{Z}}$, ${\text{M}}_{\text{D}}^{\text{X}}$, and ${\text{M}}_{\text{D}}^{\text{Z}}$).

Using the full HMDB spectral database (S3 Table), metabomatching ranked the reference metabolites for *SLC6A20*, *SLC7A9* markedly lower. Using the UMRB or BMRB spectral databases (S3 Table), metabomatching ranks the reference metabolites for *SLC6A19*, *SLC6A13*, and *PNMT* higher, but is otherwise comparable to UMDB or HMDB, respectively.

## Discussion

Under the test conditions used here, metabomatching has shown to be remarkably successful in identifying the metabolites underlying the feature associations in the investigated pseudospectra, by generally highly ranking the respective reference metabolites. In normal conditions, where the underlying metabolites are not known, the performance of metabomatching depends on the characteristics of the untargeted mGWAS.

First and foremost, metabomatching can only identify an underlying metabolite for which a spectrum is listed in the supplied spectral database. Here, we only tested metabomatching on untargeted associations that we could link to reference metabolites with listed spectra. However, both the CoLaus [17] and SHIP [20] mGWASes discovered feature associations to which metabomatching did not assign plausible candidates, likely because the spectra of the underlying metabolites are absent from HMDB.

Similarly, metabomatching can only properly rank the metabolite to identify if the NMR spectrum in the provided database does not significantly deviate from the NMR spectrum as measured in the experimental conditions specific to the mGWAS. Such deviations are common, and can be significant. For example, if we compare match sets pairs for the 318 metabolites of spectra that are listed both in HMDB and BMRB, but were not necessarily acquired under identical experimental conditions, we find that the match sets of 133 metabolites differ by at least one feature and that the match sets of 29 metabolites have no common features. Increasing the neighborhood parameter *δ* in the match set definitions can mitigate such deviations, but in turn, larger neighborhoods make metabolites generally more difficult to distinguish.

If the metabolite underlying an observed metabolome feature association is listed in the database, and if the listed spectrum does not significantly deviate from the mGWAS specific spectrum, then the underlying metabolite obtains a high metabomatching score. For the metabolite to also obtain a high rank, however, it needs to outscore other listed metabolites. If the observed feature association is strong enough, the underlying metabolite outscores all those metabolites whose spectra do not include the associated feature, and whose scores therefore rely essentially on the level of noise in the pseudospectrum. The *p*-value threshold of 5 × 10^−8^, the Bonferroni threshold for significance when correcting only for the number of tested SNPs, is usually sufficient for signal-based scores to outrank almost all noise-based scores.

The main competition for top metabomatching rank then stems from metabolites with a listed spectrum which also matches the associated feature. Therefore, the more distinctive the underlying metabolite, that is the more dissimilar it is from the other metabolites in the spectral database, the higher it ranks. The distinctiveness of a metabolite is not an intrinsic property of the metabolite spectrum, not only because it depends on the chosen database, but because it depends on the mGWAS itself. For example, small peaks that contribute to the distinctiveness of a spectrum may be lost in a low powered mGWAS, thereby making metabolites distinct in spectra indistinguishable for metabomatching. However, the strongest matches among our test cases, that is those for loci *AGXT2* and *SLC7A9* in CoLaus (Table 1), and *DAB1*, *ENTTPL*, and *AGXT2* in SHIP (Table 2), follow a trend, similar to that suggested by our simulation results: the greater the number of clusters of peaks in the spectrum of a metabolite, the greater, and more resilient, its rank. Even though not all spectrum peaks will necessarily show strong association, metabolites with high cluster count spectra do tend to produce high cluster count pseudospectra. The corresponding matches are generally characterized in both high score and high distinctiveness.

The ideal settings under which to run metabomatching are specific to every mGWAS, and depend on the experimental conditions under which the feature metabolome was acquired, the data processing applied, and the statistical power of the study. Consequently, while the default settings (1-compound, HMDB, peak-mode, *χ*^2^-scoring, *δ* = 0.03 and without decorrelation) provide a good starting point, the performance of metabomatching can be significantly improved by adapting the settings to the study.

The greatest impact on performance is likely achieved simply by selecting the appropriate biofluid-specific, and therefore smaller, spectral database. Then, it is advisable to run metabomatching with wide neighborhoods (*δ* = 0.05) first, to uncover potential issues of deviations of study spectra from reference spectra. While wide neighborhoods tend to muddle metabomatching results in general, good matches should still be obtained in specific cases where the SNP associates with multiple peaks in distinct clusters of a metabolite spectrum or with peaks of a distinctive metabolite spectrum. Guided by the performance on such cases, metabomatching should be run with progressively smaller values of *δ*, until the smallest *δ*, which still accounts for the observed deviations between study and reference spectra, is reached.

Pseudospectra should then be individually inspected for the need for multiple-compound metabomatching: 2-compound metabomatching if the associations are of the same effect direction, but no single metabolite matches them all; ±-metabomatching if the associations are of opposite effect directions.

With biofluid, *δ* and metabomatching variants defined, runs with decorrelation (taking λ = 0.5) or *Z*-scoring can be tested. Which scoring or decorrelation setting performs better is difficult to evaluate, and may be essentially subjective unless prior knowledge about the underlying metabolites is available.

Finally, metabomatching against other reference biofluid-specific subsets, such as those of BMRB or of multiplet description HMDB, may prove to provide stronger matches due to better conforming reference spectra, while metabomatching against full HMDB or BMRB may, for unmatched pseudospectra, identify metabolites that occur in the studied biofluid, while not being annotated as such.

Applying this procedure allows metabomatching to run in the settings best suited to the investigated mGWAS, and present the most likely candidate metabolites, among the provided set of reference metabolites, underlying observed SNP-feature associations. Because the spectral database never fully conforms to the set of metabolites investigated in any specific study, however, metabomatching cannot provide definitive identification. In some cases, additional evidence can strengthen metabomatching candidates, such as a direct biological connection between gene and candidate metabolite (*ENTTPL*, *DMGDH*) or coherent targeted mGWAS association results (all testable associations presented here, but *CPS1*, *AGXT2*, *SLC6A13*, and *HPD*, in particular, for which targeted association results also exist in blood and mass spectrometry mGWASes [7]). For the remaining cases, in-sample identification through manual annotation or further measurement from spiking experiments or 2-dimensional NMR spectroscopy may be required to verify the candidates provided by metabomatching.

### Conclusion

While not yet as widespread as the targeted approach, the untargeted approach to metabolome-wide genome-wide association studies has already shown compelling results. Because it analyses all measured metabolome features, the untargeted approach more fully exploits experimental data and may discover genetically determined metabolites that were missed, because they eluded identification, by a targeted approach. By focusing the identification effort on the comparatively few metabolites found to be genetically determined, the untargeted approach also presents the pragmatic advantage of shortening the path from spectral metabolome data to mGWAS results.

Metabomatching further reduces this identification effort, by combining genetic spiking information with spectral reference data to assign candidate metabolites to genetically associated metabolome features. In addition, because identification through genetic spiking is not an in-sample procedure, metabomatching becomes of particular interest when applied in an mGWAS that combines untargeted and targeted approaches. In such a combined mGWAS, metabomatching can both provide an independent line of evidence for in-sample identifications of metabolites, and inform on the identity of metabolites that were missed by the targeted approach because they eluded in-sample identification.

Naturally, while focus was placed here, and in previous applications of metabomatching, on pseudospectra resulting from genetic association with NMR features, metabomatching is not limited to genome-wide association studies. Any trait that influences, or is influenced by, metabolome features produces an association pseudospectrum to which metabomatching can assign candidates. Notably, metabolome-wide association studies, analyzing the effects of the metabolome on organismal traits, would similarly benefit from both the untargeted approach and metabomatching.

The performance of metabomatching is inherently linked to the strength of genetic spiking and the quality of spectral databases. With increasing mGWAS sample sizes, and the continuing efforts to establish spectral databases that are more complete and better annotated, both conditions are expected to improve. Metabomatching is therefore not only likely to become a valuable tool for exploring the links to metabolites of listed spectrum, but may also provide impetus to complete databases of spectral information for human metabolites, reducing instances where no good match can be found.

### Software

Metabomatching is written for Matlab and compatible with octave. Documentation and code can be obtained from the metabomatching website http://www.unil.ch/cbg/index.php?title=metabomatching or GitHub. Metabomatching is also available as a docker container, and within the metabolomics e-infrastructure PhenoMeNal http://phenomenal-h2020.eu.

## Supporting information

S1 Table CoLaus metabomatching results for different values of the shrinkage parameter λ. Decorrelation has only a minor effect on metabomatching rankings. For λ ∈ [0.1, 0.9] only the *HPD*-*α*-hydroxyisobutyrate rank is significantly affected, going to 1 from 4 (at λ = 1). Without any shrinkage (λ = 0), however, several metabolites acquire artificially high scores, leading to lower ranks of the control metabolites for *SLC6A20* and *UPS9* in both peak- and multiplet-mode.
(PDF)

S2 Table CoLaus metabomatching results against HMDB, UMRB, and BMRB spectral databases. Metabomatching performance using the spectral reference database UMRB, that is the urine-specific subset of BMRB, is similar to the performance using the spectral reference database UMDB. Trimethylamine ranks higher for *PYROXD2*, because the competing metabolites score lower in UMRB than UMDB. Tyrosine ranks lower because the BMRB listed spectrum deviates more from its pseudospectrum-implied CoLaus spectrum than the HMDB spectrum does. *α*-hydroxyisobutyrate and 3-hydroxyisovalerate do not have spectra listed in BMRB. Using the full HMDB or BMRB databases introduces more competing metabolites, significantly affecting the ranks of *PYROXD2* and *PNMT*.
(PDF)

S3 Table SHIP metabomatching results against HMDB, UMRB, and BMRB spectral databases. We see that the spectra listed in BMRB tend to correspond better, overall, to the pseudospectrum-implied spectra of the metabolites in SHIP, resulting in better metabomatching ranks. This applies in particular to histidine, tyrosine, and 3-aminoisobutyrate, resulting in significantly better ranks for *SLC6A19*, *SLC6A13*, and *PNMT*. Strong matches in *DAB1*, *SLC6A20*, *ENTPPL*, *AGXT2*, *SLC36A2*, and *NAT2* maintain their high ranks when metabomatching is run against the full databases HMDB and BMRB. The ranks of weaker matches, which already suffered strong competition when using the urine-specific subsets, drop; in cases such as *SLC6A20* and *SLC7A9*, significantly so.
(PDF)

S1 Fig CoLaus metabomatching figures. Full results for UMDB peak-mode *χ*^2^-scoring metabomatching for each of the nine testable CoLaus pseudospectra. Multiplet-mode metabomatching results are shown only for *HPD*, where they differ notably from peak-mode metabomatching results. The navigation table in the footer allows direct access to a specific pseudospectrum. The reference metabolite is indicated by a hash mark (#) next to its name.
(PDF)

S2 Fig SHIP metabomatching figures. Full results for UMDB peak-mode *χ*^2^-scoring metabomatching for each of the fourteen testable SHIP pseudospectra. 2-compound metabomatching results are shown for *CPS1* and *HPD*, and ±-metabomatching results for *NAT2*. Multiplet-mode metabomatching results are shown only where they differ notably from peak-mode metabomatching results, that is for *CPS1*, *XYLB*, and *HPD*. The navigation table in the footer allows direct access to a specific pseudospectrum. The table is repeated on subsequent pages. The reference metabolites are marked with a hash mark (#) next to their name. To maintain a consistent layout where necessary, long metabolite names are replaced by the metabolite chemical abstract service registry number (CASRN), and a conversion table added to the bottom of the figure.
(PDF)

S3 Fig An example of the effect of decorrelation in metabomatching. The pseudospectrum of rs10774021 in *SLC6A13* is characterized by a single significantly associated feature: feature 1.19. (A) With *χ*^2^-scoring metabomatching, the association with feature 1.19 is sufficient for metabolites of spectrum matching feature 1.19, including the reference metabolite 3-aminoisobutyrate, to obtain top ranks. (B) With *Z*-scoring metabomatching, metabolites of spectrum matching 1.19 are outranked by metabolites matching peaks in the region between 3.6 and 3.9 ppm, which produces, on its own, a score of 11.8. (C) The 3.6 to 3.9 ppm region is characterized by strong correlation. By applying decorrelation, with λ = 0.5, the score produced by the region, on its own, is reduced to 0.8. Correspondingly, with decorrelation, 3-aminoisobutyrate outranks most metabolites that, without decorrelation, ranked highly by matching the 3.6 to 3.9 ppm region.
(PDF)

S4 FigSimulation results for metabolome model including genetic noise.Median $r_{90}^{m}$, with metabolites grouped by their rounded size, and from light to dark blue, *β* = 0.2, 0.4, 1.6. We show results for UMDB and HMDB, neighborhood parameters *δ* = 0.02 and 0.05, *χ*^2^- and *Z*-scoring, and two genetic noise levels, defined by *N*_*a*_ and *α* set to 16 and 0.4, and 64 and 0.6, respectively. From these cases, we see that the performance of metabomatching is consistently stronger in the smaller spectral database UMDB, and for smaller *δ*. For the weaker genetic noise, the median $r_{90}^{m}$ is equal to, or close to, 1, for sufficiently large *β*, except in the case of *δ* = 0.05 in HMDB for *s*^*m*^ = 2. For strong genetic noise, metabomatching performance is consistently poorer, with $r_{90}^{m}$ often far from 1. *χ*^2^-scoring performs better than *Z*-scoring under weak genetic noise. When genetic noise is strong, however, *Z*-scoring performs almost invariably better: when *β* is large, there are sizes *s*^*m*^ for which *Z*-scoring produces $r_{90}^{m}$ close to 1 while *χ*^2^-scoring fails to do so.(PDF)
