# Supplementary material for: Metabomatching: Using genetic association to identify metabolites in proton NMR spectroscopy
Source: PLoS Comput Biol. 2017 Dec 1;13(12):e1005839. doi: 10.1371/journal.pcbi.1005839 (PMC5711027; doi:10.1371/journal.pcbi.1005839)
Supplement: S1 Table — Decorrelation has only a minor effect on metabomatching rankings. For λ ∈ [0.1, 0.9] only the HPD-α-hydroxyisobutyrate rank is significantly affected, going to 1 from 4 (at λ = 1). Without any shrinkage (λ = 0), however, several metabolites acquire artificially high scores, leading to lower ranks of the control metabolites for SLC6A20 and UPS9 in both peak- and multiplet-mode. (PDF) [file pcbi.1005839.s001.pdf]

		mode	P					M				
		λ	1.0	0.9	0.5	0.1	0.0	1.0	0.9	0.5	0.1	0.0
SLC6A20	dimethylglycine		2	1	1	1	6	2	2	1	2	4
AGXT2	3-aminoisobutyrate		1	1	1	1	1	1	1	1	1	1
SOSTDC1	taurine		□	□	□	□	□	□	□	□	14	□
PYROXD2	trimethylamine		5	5	4	7	7	2	2	4	2	4
SLC6A13	3-aminoisobutyrate		4	4	4	3	5	1	1	4	1	2
HPD	α-hydroxyisobutyrate		4	1	1	1	3					
HPD	3-hydroxyisovalerate							1	1	1	2	8
PNMT	tyrosine		3	3	3	2	2	1	1	3	1	1
SLC7A9	lysine		1	1	1	1	1	1	1	1	1	1
UPS9	sucrose		1	2	2	2	8	1	1	2	2	5
