# Supplementary material for: Metabomatching: Using genetic association to identify metabolites in proton NMR spectroscopy
Source: PLoS Comput Biol. 2017 Dec 1;13(12):e1005839. doi: 10.1371/journal.pcbi.1005839 (PMC5711027; doi:10.1371/journal.pcbi.1005839)
Supplement: S2 Table — Metabomatching performance using the spectral reference database UMRB, that is the urine-specific subset of BMRB, is similar to the performance using the spectral reference database UMDB. Trimethylamine ranks higher for PYROXD2, because the competing metabolites score lower in UMRB than UMDB. Tyrosine ranks lower because the BMRB listed spectrum deviates more from its pseudospectrum-implied CoLaus spectrum than the HMDB spectrum does. α-hydroxyisobutyrate and 3-hydroxyisovalerate do not have spectra listed in BMRB. Using the full HMDB or BMRB databases introduces more competing metabolites, significantly affecting the ranks of PYROXD2 and PNMT. (PDF) [file pcbi.1005839.s002.pdf]

	database	HMDB				UMRB		BMRB	
	mode	P	P	M	M	P	P	P	P
	λ	1.0	0.5	1.0	0.5	1.0	0.5	1.0	0.5
SLC6A20	dimethylglycine	2	2	2	2	2	1	4	1
AGXT2	3-aminoisobutyrate	1	1	1	1	1	1	1	1
SOSTDC1	taurine	□	□	□	□	□	□	□	□
PYROXD2	trimethylamine	□	□	15	9	1	1	7	4
SLC6A13	3-aminoisobutyrate	5	9	1	2	2	3	2	3
HPD	α-hydroxyisobutyrate	□	1						
HPD	3-hydroxyisovalerate			3	16				
PNMT	tyrosine	15	13	10	4	□	□	□	□
SLC7A9	lysine	2	2	2	4	1	1	1	4
UPS9	sucrose	5	2	7	1	1	2	3	3
