# Supplementary material for: Metabomatching: Using genetic association to identify metabolites in proton NMR spectroscopy
Source: PLoS Comput Biol. 2017 Dec 1;13(12):e1005839. doi: 10.1371/journal.pcbi.1005839 (PMC5711027; doi:10.1371/journal.pcbi.1005839)
Supplement: S3 Table — We see that the spectra listed in BMRB tend to correspond better, overall, to the pseudospectrum-implied spectra of the metabolites in SHIP, resulting in better metabomatching ranks. This applies in particular to histidine, tyrosine, and 3-aminoisobutyrate, resulting in significantly better ranks for SLC6A19, SLC6A13, and PNMT. Strong matches in DAB1, SLC6A20, ENTPPL, AGXT2, SLC36A2, and NAT2 maintain their high ranks when metabomatching is run against the full databases HMDB and BMRB. The ranks of weaker matches, which already suffered strong competition when using the urine-specific subsets, drop; in cases such as SLC6A20 and SLC7A9, significantly so. (PDF) [file pcbi.1005839.s003.pdf]

Locus	Control Metabolite	HMDB P χ^2	HMDB P Z	HMDB M χ^2	HMDB M Z	UMRB P χ^2	UMRB P Z	BMRB P χ^2	BMRB P Z
DAB1	hippurate	1	1	1	2	1	1	1	1
CPS1	glycine	□	□	□	□	7	□	17	□
CPS1	creatine	6	□	□	□	4	□	8	□
CPS1	creatine & glycine	5	□	□	□	3	□	4	□
XYLB	glycolate	2	□	□	□	2	□	2	□
SLC6A20	dimethylglycine	2	5	2	3	1	1	1	3
ENTPPL	ethanolamine	1	7	1	□	1	1	1	4
SLC6A19	histidine	□	□	12	□	2	3	10	□
SLC6A19	tyrosine	□	□	13	□	4	7	□	□
SLC6A19	histidine & tyrosine	□	□	□	□	□	□	□	□
AGXT2	3-aminoisobutyrate	1	3	1	4	1	1	1	1
DMGDH	dimethylglycine	13	□	14	□	3	□	17	□
SLC36A2	glycine	1	5	5	13	1	2	1	4
NAT2	formate	□	3	□	1	□	2	□	3
NAT2	formate & any (±)	□	1	2	1	□	1	1	1
SLC6A13	3-aminoisobutyrate	11	18	□	□	2	1	3	7
HPD	α-hydroxyisobutyrate	□	9	□	3	×	×	×	×
HPD	3-hydroxyisovalerate	□	□	□	□	×	×	×	×
HPD	···butyrate & ···valerate	□	□	□	□	×	×	×	×
PNMT	tyrosine	□	□	□	□	1	12	□	□
PNMT	histidine	□	□	□	□	6	□	□	□
PNMT	alanine	□	□	□	□	14	15	□	□
SCL7A9	lysine	15	□	□	□	11	□	□	□

(Header rows: Database / Mode / Scoring.)
