# Supplementary material for: Metabomatching: Using genetic association to identify metabolites in proton NMR spectroscopy
Source: PLoS Comput Biol. 2017 Dec 1;13(12):e1005839. doi: 10.1371/journal.pcbi.1005839 (PMC5711027; doi:10.1371/journal.pcbi.1005839)
Supplement: S1 Fig — Full results for UMDB peak-mode χ2-scoring metabomatching for each of the nine testable CoLaus pseudospectra. Multiplet-mode metabomatching results are shown only for HPD, where they differ notably from peak-mode metabomatching results. The navigation table in the footer allows direct access to a specific pseudospectrum. The reference metabolite is indicated by a hash mark (#) next to its name. (PDF) [file pcbi.1005839.s004.pdf]

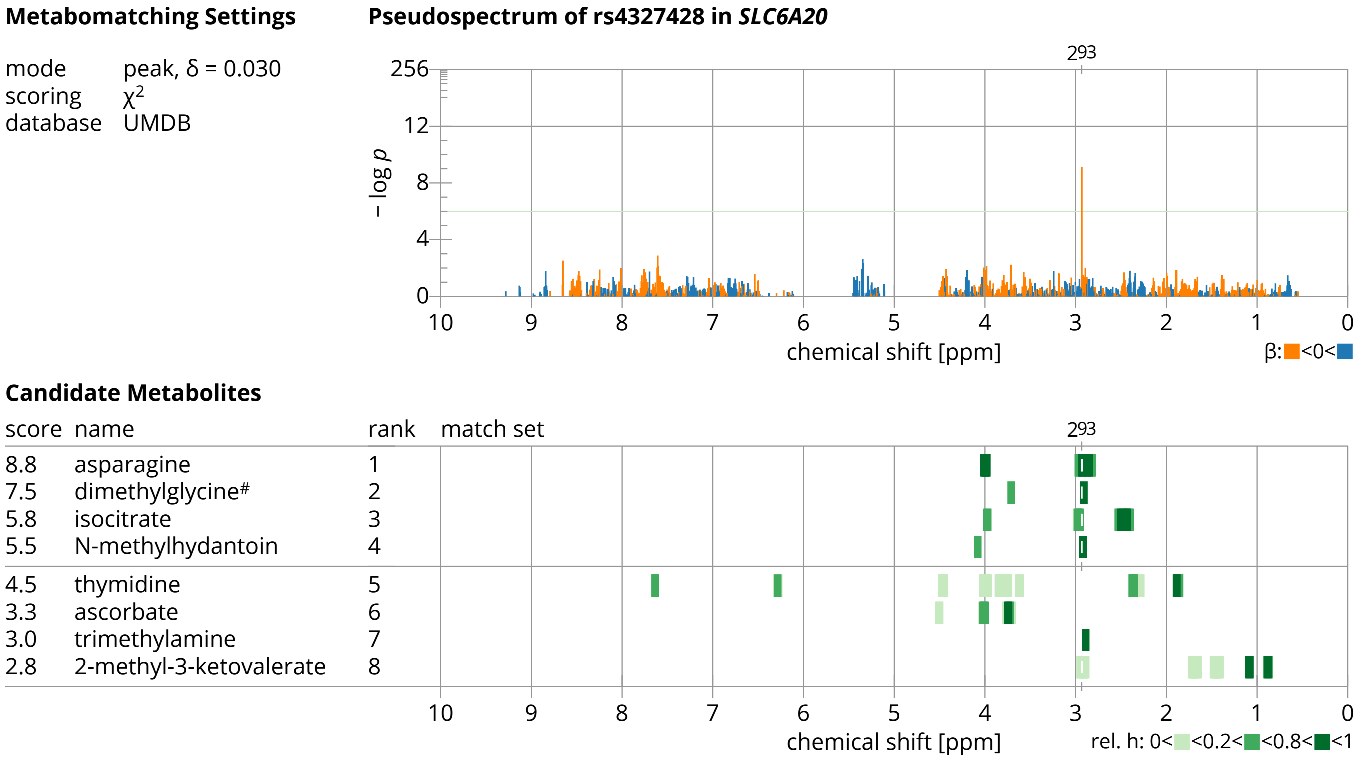

(A) CoLaus, *SLC6A20*. Peak-mode, χ^2-scoring metabomatching. The rs4327428 pseudospectrum is characterized by a single significantly associated feature: feature 2.93. Dimethylglycine and N-methylhydantoin spectra produce the best, though not top-ranked, matches. For both, the highest spectrum peak matches feature 2.93, and the second peak is unmatched. While trimethylamine produces a strong one-to-one match, feature 2.93 falls on the very edge of the peak neighbourhood.

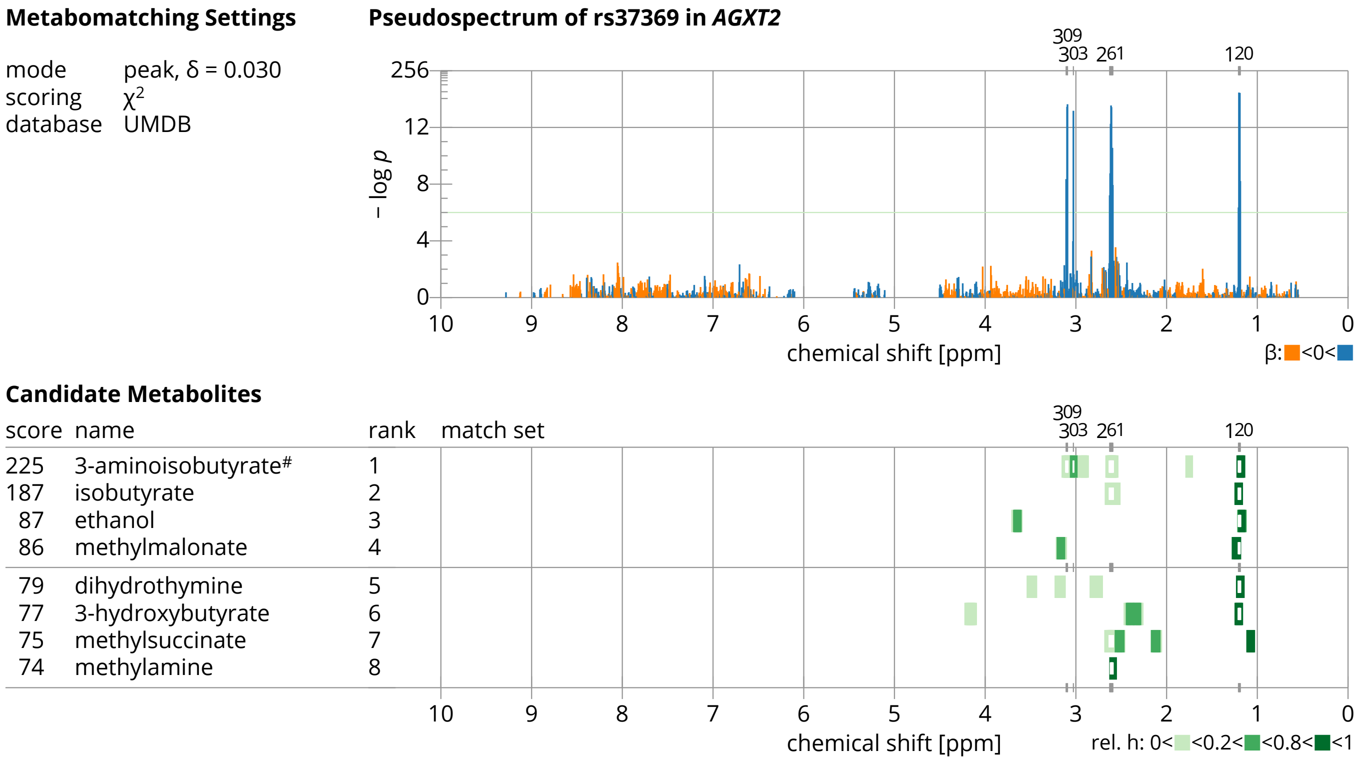

(B) CoLaus, *AGXT2*. Peak-mode, χ^2-scoring metabomatching. The rs37369 pseudospectrum is characterized by four significantly associated feature clusters, with equal effect directions. The 3-aminoisobutyrate spectrum produces a strong match, with three peak clusters matching all four significantly associated feature clusters.

Navigation:

CoLaus	*SLC6A20*	*AGXT2*	*SOSTDC1*	*PYROXD2*	*SLC6A13*	*HPD*	*HPD/M*	*PNMT*	*SLC7A9*	*UPS9*	
SHIP	*DAB1*	*CPS1*	*CPS1/2c*	*CPS1/M*	*CPS1/M/2c*	*XYLB*	*XYLB/M*	*SLC6A20*	*ENTPPL*	*SLC6A19*	*AGXT2*
	DMGDH	*SLC36A2*	*NAT2*	*NAT2/±*	*SLC6A13*	*HPD*	*HPD/2c*	*HPD/M*	*HPD/M/2c*	*PNMT*	*SLC7A9*

Metabomatching Settings

mode	peak, δ = 0.030
scoring	χ^2
database	UMDB

Pseudospectrum of rs17169536 in *SOSTDC1*

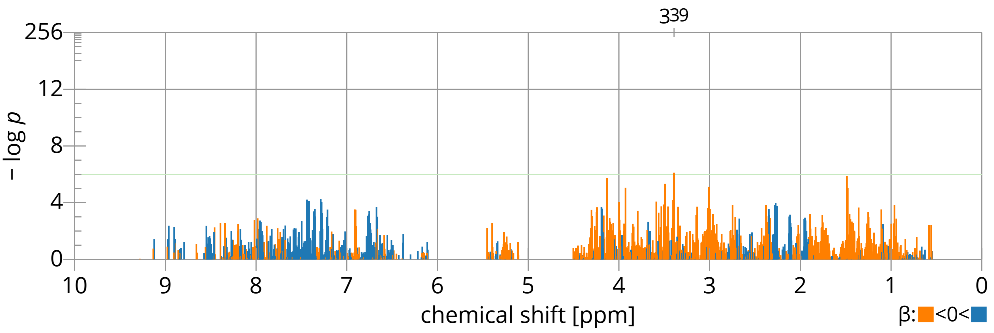

Candidate Metabolites

score	name	rank
53.2	aminoadipate	1
51.4	lysine	2
49.6	sucrose	3
49.1	2-methylbutyrylglycine	4
42.8	N-acetylneuraminate	5
42.4	thymidine	6
41.7	3-methyladipate	7
13.3	taurine#	93

match set

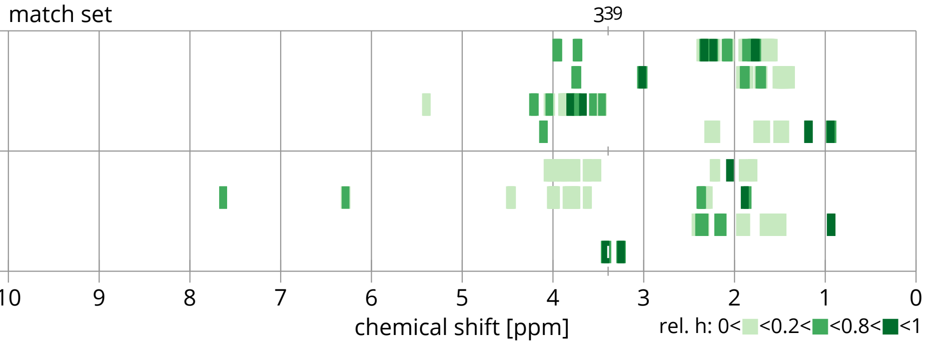

(C) CoLaus, *SOSTDC1*. Peak-mode, χ^2-scoring metabomatching. The rs17169536 pseudospectrum shows inflation, with multiple feature regions approaching the significance threshold of 10^{-6}. This inflation produces high scores for many metabolites in the spectral database, so that, even though one of the taurine spectrum peaks matches feature 3.39 specifically, metabomatching does not rank taurine a plausible candidate.

Metabomatching Settings

mode	peak, δ = 0.030
scoring	χ^2
database	UMDB

Pseudospectrum of rs4488133 in *PYROXD2*

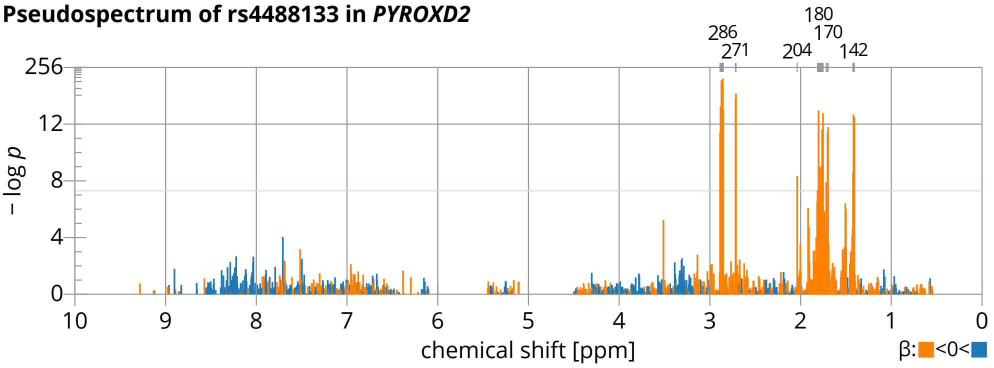

Candidate Metabolites

score	name	rank
298	asparagine	1
283	hydroxyphenyllactate	2
261	2-methyl-3-ketovalerate	3
260	3-methyl-2-oxovalerate	4
183	trimethylamine#	5
173	3-aminoisobutyrate	6
162	lysine	7
149	methylguanidine	8

match set

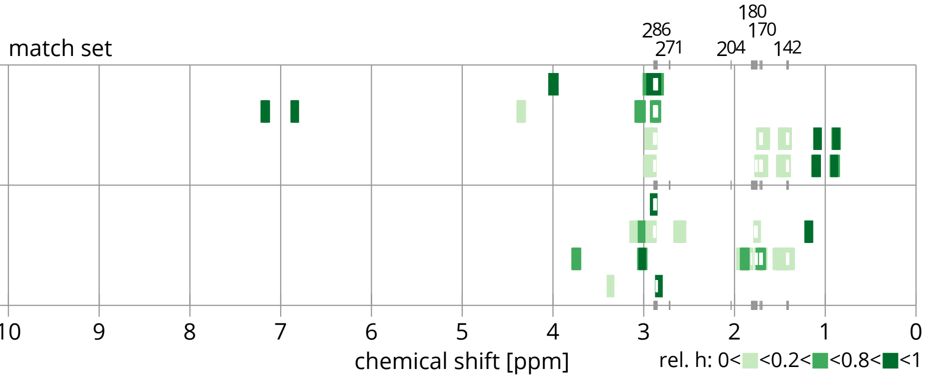

(D) CoLaus, *PYROXD2*. Peak-mode, χ^2-scoring metabomatching. The rs4488133 pseudospectrum is characterized by six significantly associated features or feature clusters, with equal effect directions. Of these, feature 2.86 shows the strongest association, and is matched by the single peak of the trimethylamine spectrum. The pseudospectrum suggests the presence of least one additional underlying metabolite, to match the five other significantly associated feature clusters.

Navigation:

CoLaus	*SLC6A20*	*AGXT2*	*SOSTDC1*	*PYROXD2*	*SLC6A13*	*HPD*	*HPD/M*	*PNMT*	*SLC7A9*	*UPS9*
SHIP	*DAB1*	*CPS1*	*CPS1/2c*	*CPS1/M*	*CPS1/M/2c*	*XYLB*	*XYLB/M*	*SLC6A20*	*ENTPPL*	*SLC6A19*
	DMGDH	*SLC36A2*	*NAT2*	*NAT2/±*	*SLC6A13*	*HPD*	*HPD/2c*	*HPD/M*	*HPD/M/2c*	*PNMT*

Metabomatching Settings

mode	peak, δ = 0.030
scoring	χ^2
database	UMDB

Pseudospectrum of rs10774021 in *SLC6A13*

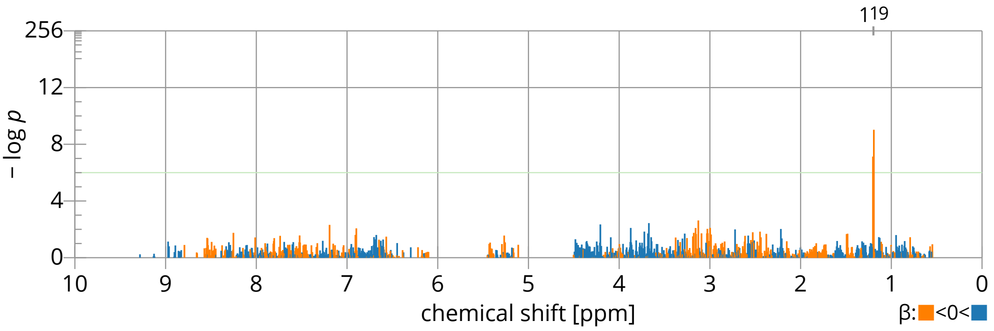

Candidate Metabolites

score	name	rank
10.9	methylmalonate	1
9.5	isobutyrate	2
9.2	ethanol	3
8.6	3-aminoisobutyrate#	4
5.5	dihydrothymine	5
5.4	fucose	6
4.9	3-hydroxybutyrate	7
3.5	isoleucine	8

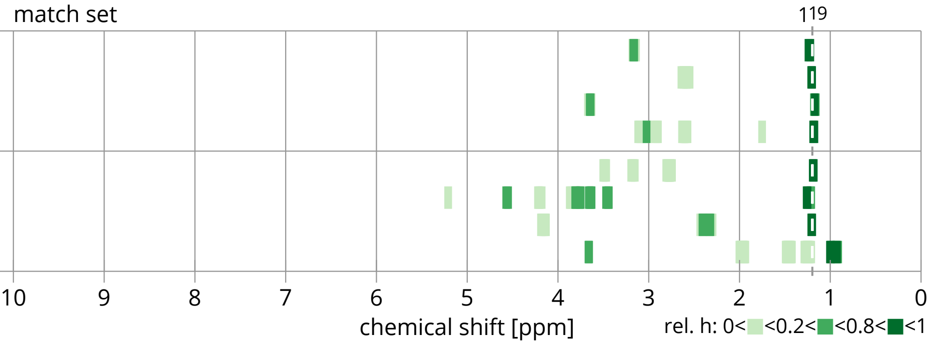

(E) CoLaus, *SLC6A13*. Peak-mode, χ^2-scoring metabomatching. The rs10774021 pseudospectrum is characterized by a cluster of significantly associated features, led by feature 1.19. The five top-ranked metabolites match feature 1.19, but have other, unmatched, peaks, and are equally likely candidates. Compare with the *AGXT2* pseudospectrum (Fig S1B), where the same feature cluster (led by feature 1.20) is only one of four significantly associated clusters, and where the match to underlying 3-aminoisobutyrate is clear.

Metabomatching Settings

mode	peak, δ = 0.030
scoring	χ^2
database	UMDB

Pseudospectrum of rs7314056 in *HPD*

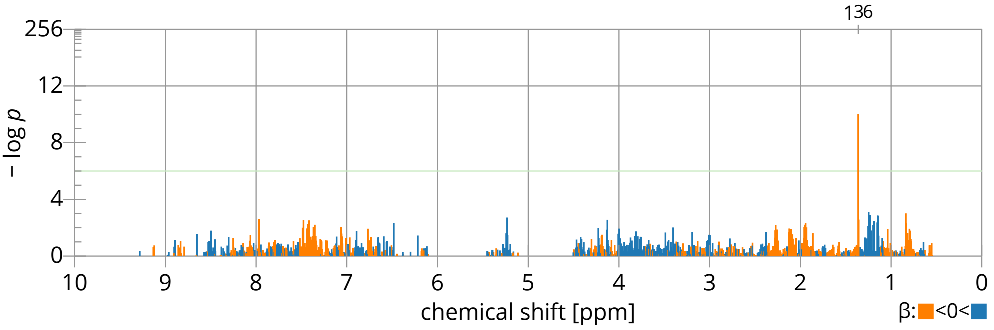

Candidate Metabolites

score	name	rank
10.6	mandelate	1
10.3	fucose	2
9.3	cinnamate	3
7.1	α-hydroxyisobutyrate#	4
6.3	quinolinate	5
6.2	methylmalonate	6
5.7	3-hydroxybutyrate	7
5.5	3-hydroxyisovalerate#	9

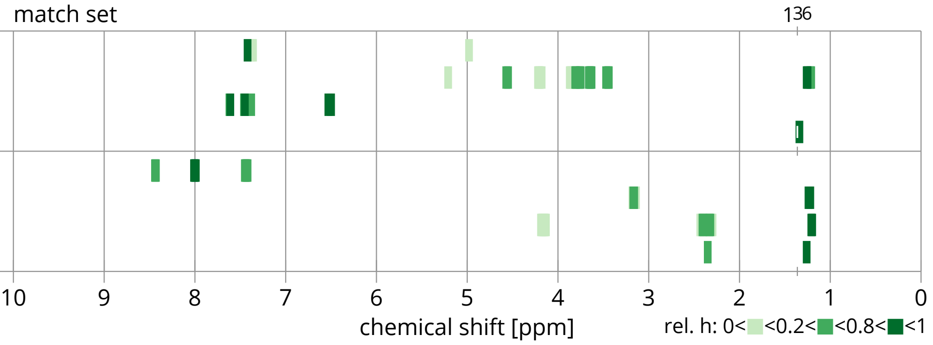

(F) CoLaus, *HPD*. Peak-mode, χ^2-scoring metabomatching. The rs7314056 pseudospectrum is characterized by a single significantly associated feature: feature 1.36. α-hydroxyisobutyrate produces a strong one-to-one match, and is the most likely candidate. The three top ranked metabolites achieve their score by a fortunate selection of pseudospectrum features, but are not tenable candidates. The second known association in this locus is with 3-hydroxyisovalerate. Because the peak description match set does not contain feature 1.36, 3-hydroxyisovalerate is not testable in peak-mode metabomatching. In multiplet-mode, however, this situation is reversed (see Fig S1G)

Navigation:

CoLaus	*SLC6A20*	*AGXT2*	*SOSTDC1*	*PYROXD2*	*SLC6A13*	*HPD*	*HPD/M*	*PNMT*	*SLC7A9*	*UPS9*
SHIP	*DAB1*	*CPS1*	*CPS1/2c*	*CPS1/M*	*CPS1/M/2c*	*XYLB*	*XYLB/M*	*SLC6A20*	*ENTPPL*	*SLC6A19*	*AGXT2*
	DMGDH	*SLC36A2*	*NAT2*	*NAT2/±*	*SLC6A13*	*HPD*	*HPD/2c*	*HPD/M*	*HPD/M/2c*	*PNMT*	*SLC7A9*

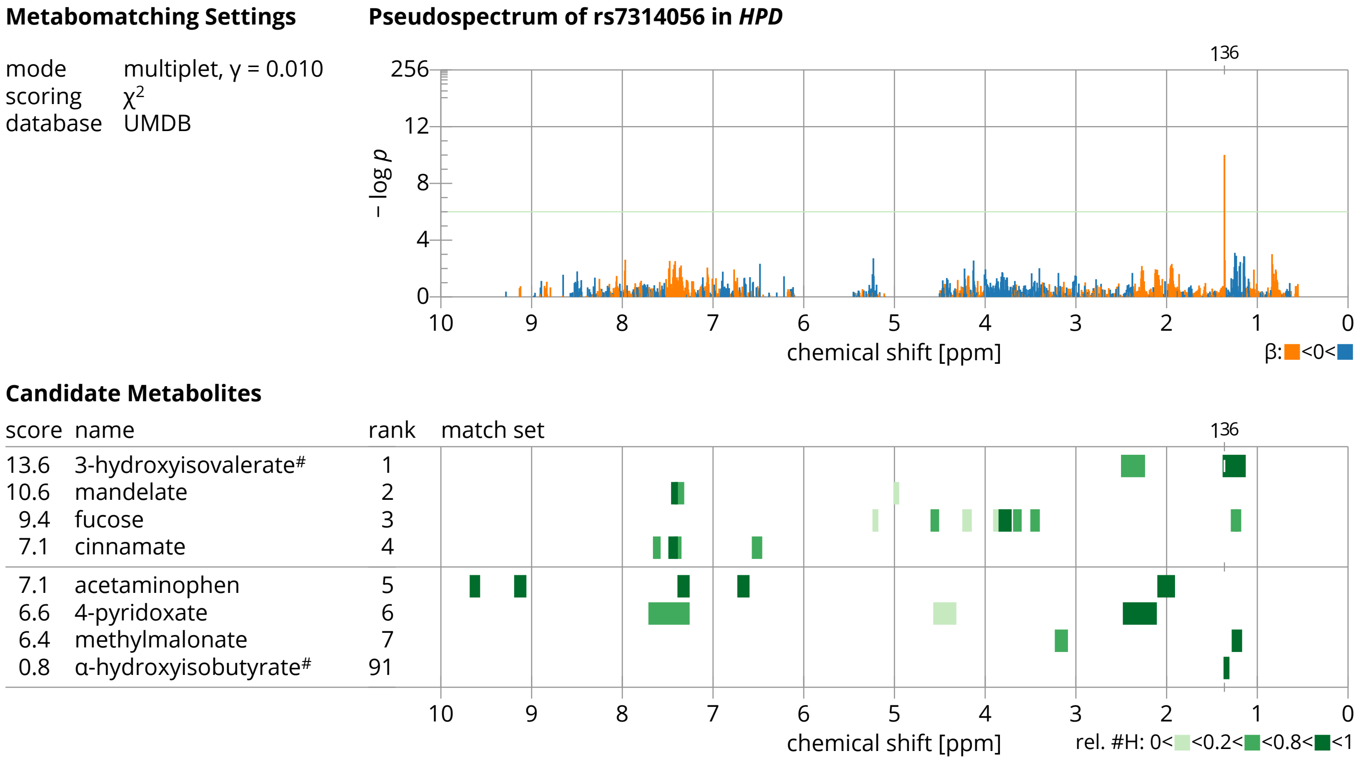

(G) CoLaus, *HPD*. Multiplet-mode, χ^2-scoring metabomatching. The rs7314056 pseudospectrum is characterized by a single significantly associated feature: feature 1.36. As seen in Fig S1F, feature 1.36 matches the spectrum of α-hydroxyisobutyrate, if considering the peak description. The multiplet range in the multiplet description of the spectrum of α-hydroxyisobutyrate narrowly excludes 1.36, however, making α-hydroxyisobutyrate not testable in multiplet mode. Conversely, the multiplet range enclosing peak 1.25 in the multiplet description of the spectrum of 3-hydroxyisovalerate is wide, and narrowly includes feature 1.36. As a result, 3-hydroxyvalerate is not only testable in multiplet mode, but achieves rank 1.

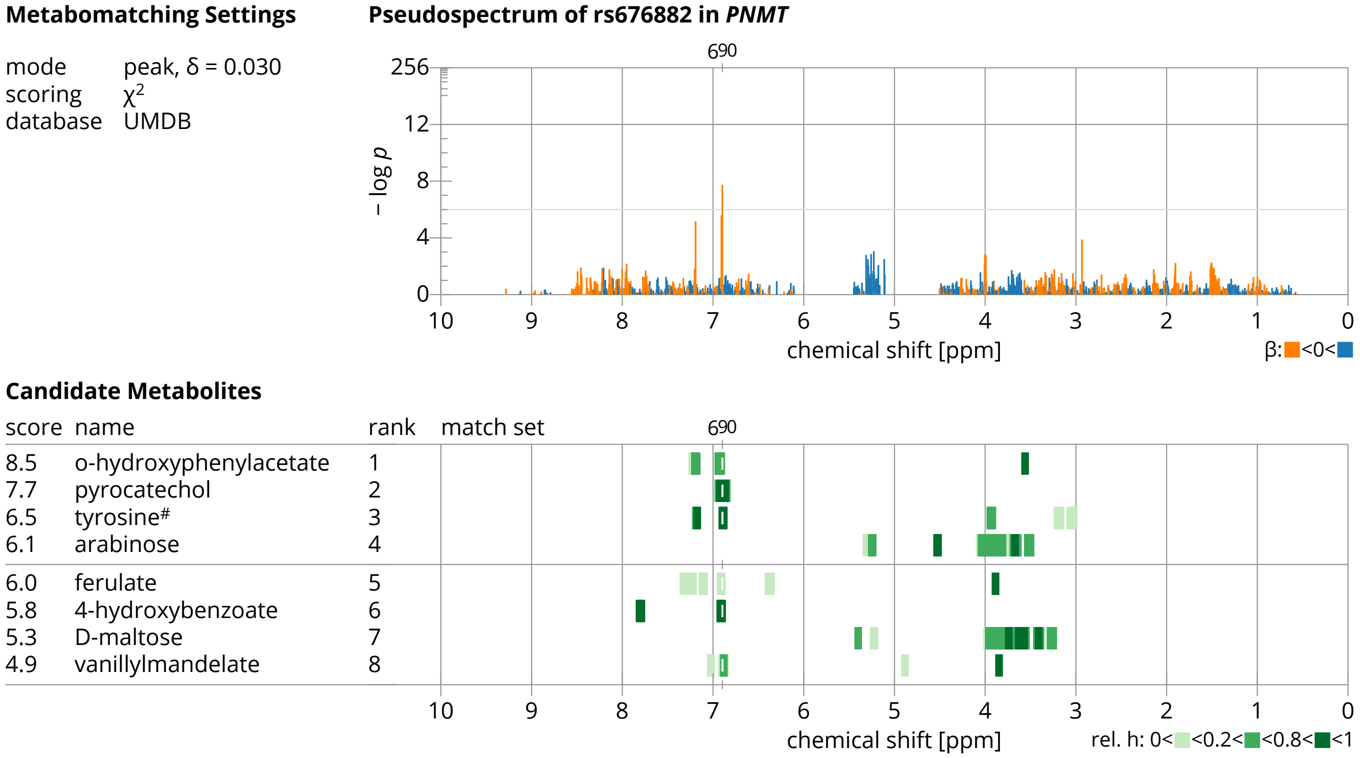

(H) CoLaus, *PNMT*. Peak-mode, χ^2-scoring metabomatching. The rs676882 pseudospectrum is characterized by a single significantly associated feature (feature 6.90) and several features of association above background but below significance. The two highest peaks of the tyrosine spectrum match both feature 6.90 and one of the secondary features.

Navigation:

CoLaus	*SLC6A20*	*AGXT2*	*SOSTDC1*	*PYROXD2*	*SLC6A13*	*HPD*	*HPD/M*	*PNMT*	*SLC7A9*	*UPS9*
SHIP	*DAB1*	*CPS1*	*CPS1/2c*	*CPS1/M*	*CPS1/M/2c*	*XYLB*	*XYLB/M*	*SLC6A20*	*ENTPPL*	*SLC6A19*	*AGXT2*
	DMGDH	*SLC36A2*	*NAT2*	*NAT2/±*	*SLC6A13*	*HPD*	*HPD/2c*	*HPD/M*	*HPD/M/2c*	*PNMT*	*SLC7A9*

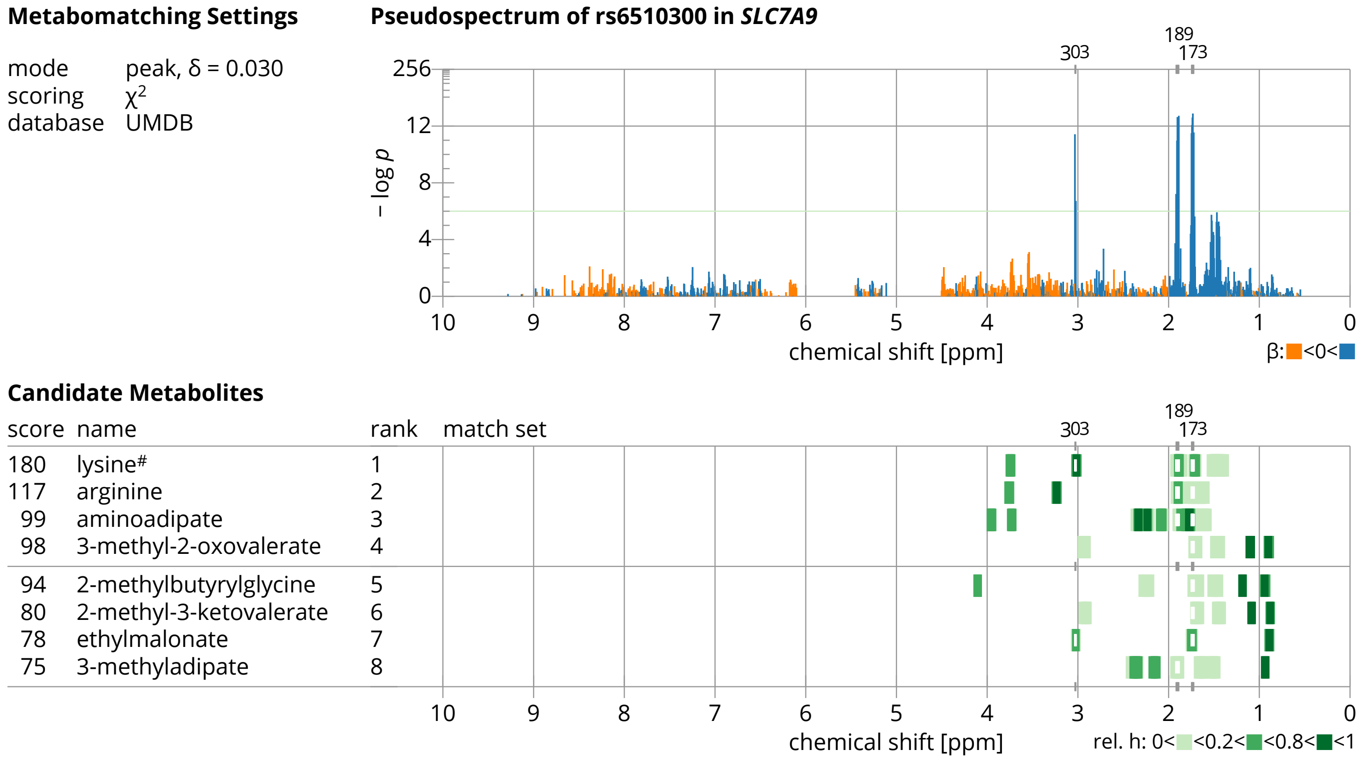

(I) CoLaus, *SLC7A9*. Peak-mode, χ^2-scoring metabomatching. The rs6510300 pseudospectrum is characterized by three significantly associated feature clusters and two almost significantly associated feature clusters, with equal effect directions. The lysine spectrum produces a strong match, with four peak clusters matching all significantly and almost significantly associated feature clusters.

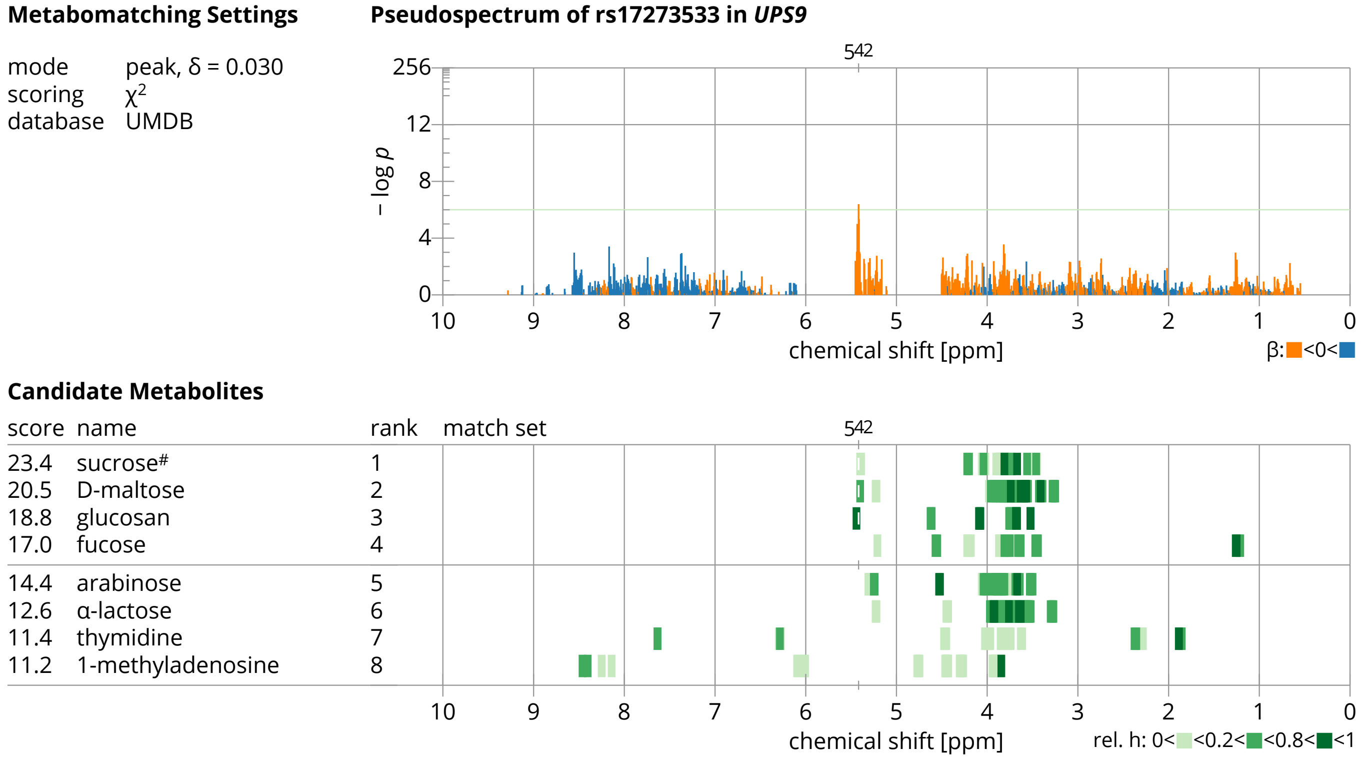

(J) CoLaus, *UPS9*. Peak-mode, χ^2-scoring metabomatching. The rs17273533 pseudospectrum is characterized by a single significantly associated feature: feature 5.42. The sucrose spectrum contains a peak matching feature 5.42, but also many others not showing association in the pseudospectrum. Therefore, while sucrose is the most likely candidate, it is not a strong one.

Navigation:

CoLaus	*SLC6A20*	*AGXT2*	*SOSTDC1*	*PYROXD2*	*SLC6A13*	*HPD*	*HPD/M*	*PNMT*	*SLC7A9*	*UPS9*	
SHIP	*DAB1*	*CPS1*	*CPS1/2c*	*CPS1/M*	*CPS1/M/2c*	*XYLB*	*XYLB/M*	*SLC6A20*	*ENTPPL*	*SLC6A19*	*AGXT2*
	DMGDH	*SLC36A2*	*NAT2*	*NAT2/±*	*SLC6A13*	*HPD*	*HPD/2c*	*HPD/M*	*HPD/M/2c*	*PNMT*	*SLC7A9*
