# Supplementary material for: Metabomatching: Using genetic association to identify metabolites in proton NMR spectroscopy
Source: PLoS Comput Biol. 2017 Dec 1;13(12):e1005839. doi: 10.1371/journal.pcbi.1005839 (PMC5711027; doi:10.1371/journal.pcbi.1005839)
Supplement: S2 Fig — Full results for UMDB peak-mode χ2-scoring metabomatching for each of the fourteen testable SHIP pseudospectra. 2-compound metabomatching results are shown for CPS1 and HPD, and ±-metabomatching results for NAT2. Multiplet-mode metabomatching results are shown only where they differ notably from peak-mode metabomatching results, that is for CPS1, XYLB, and HPD. The navigation table in the footer allows direct access to a specific pseudospectrum. The table is repeated on subsequent pages. The reference metabolites are marked with a hash mark (#) next to their name. To maintain a consistent layout where necessary, long metabolite names are replaced by the metabolite chemical abstract service registry number (CASRN), and a conversion table added to the bottom of the figure. (PDF) [file pcbi.1005839.s005.pdf]

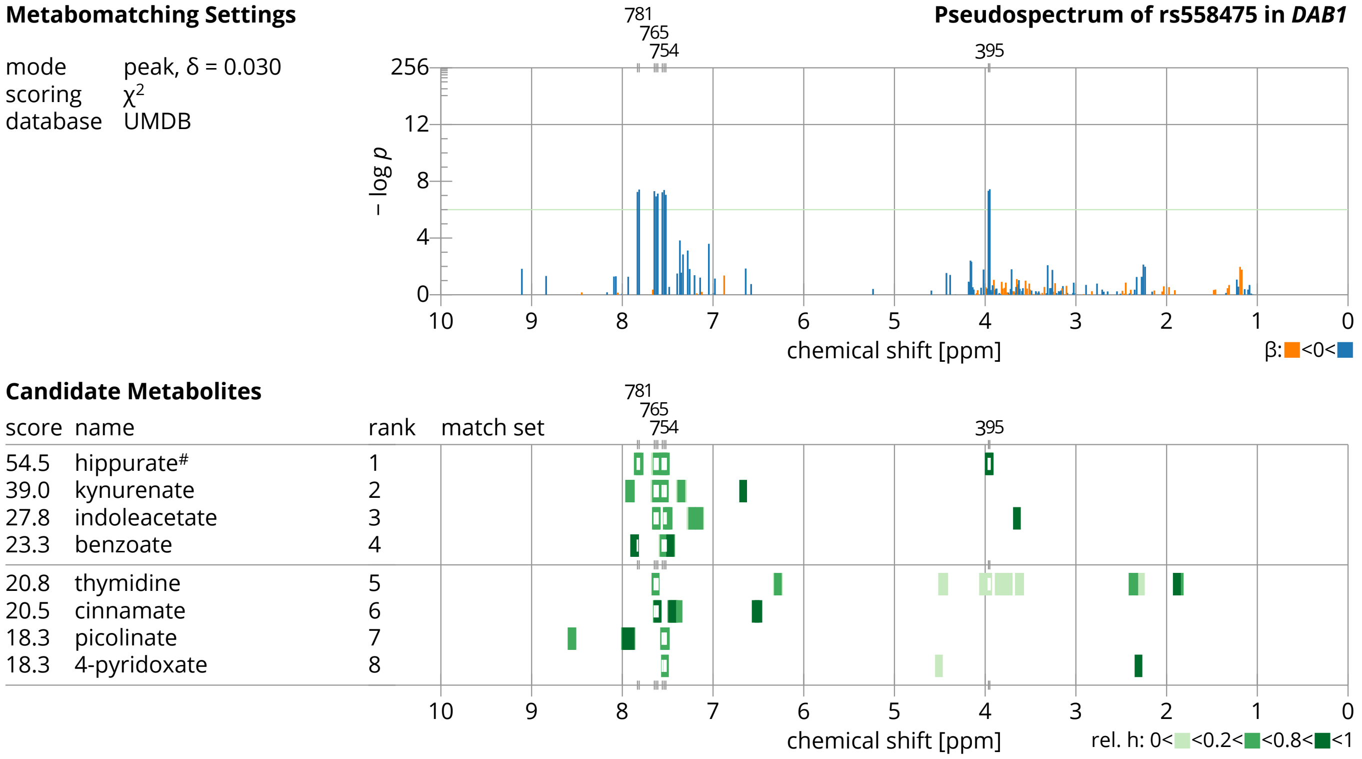

(A) SHIP, *DAB1*. Peak-mode, χ^2-scoring metabomatching. The rs558475 pseudospectrum is characterized by four clusters of significantly associated features. These feature clusters mirror the NMR spectrum of hippurate perfectly, and produce the strongest metabomatching match of all investigated pseudospectra.

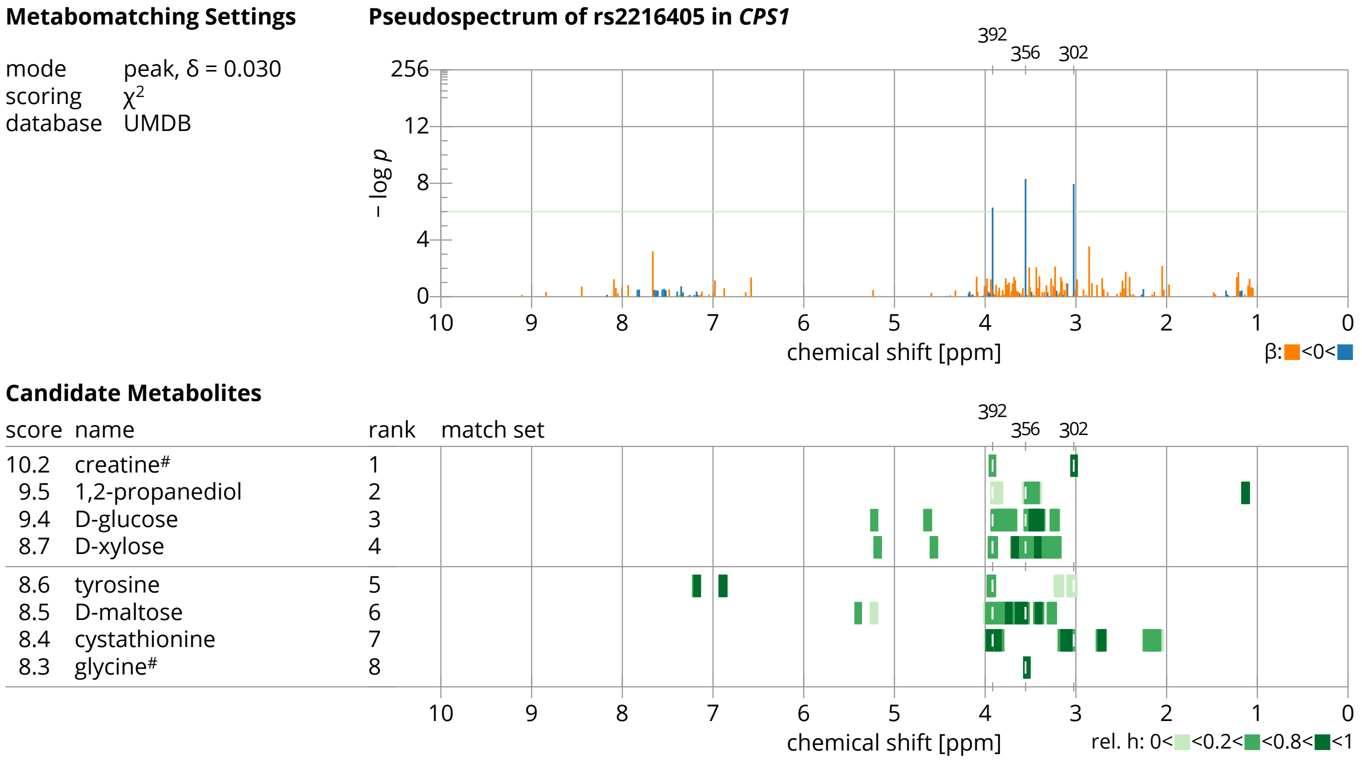

(B) SHIP, *CPS1*. Peak-mode, χ^2-scoring metabomatching. The rs2216405 pseudospectrum is characterized by three significantly associated features. These correspond perfectly to creatine and glycine. Given the presence of two reference metabolites, we run 2-compound metabomatching (see Fig S2C, *CPS1*/2c). Metabomatching in multiplet mode gives significantly different results (see Fig S2D, *CPS1*/M)

Navigation:

CoLaus	*SLC6A20*	*AGXT2*	*SOSTDC1*	*PYROXD2*	*SLC6A13*	*HPD*	*HPD/M*	*PNMT*	*SLC7A9*	*UPS9*	
SHIP	*DAB1*	*CPS1*	*CPS1/2c*	*CPS1/M*	*CPS1/M/2c*	*XYLB*	*XYLB/M*	*SLC6A20*	*ENTPPL*	*SLC6A19*	*AGXT2*
	DMGDH	*SLC36A2*	*NAT2*	*NAT2/±*	*SLC6A13*	*HPD*	*HPD/2c*	*HPD/M*	*HPD/M/2c*	*PNMT*	*SLC7A9*

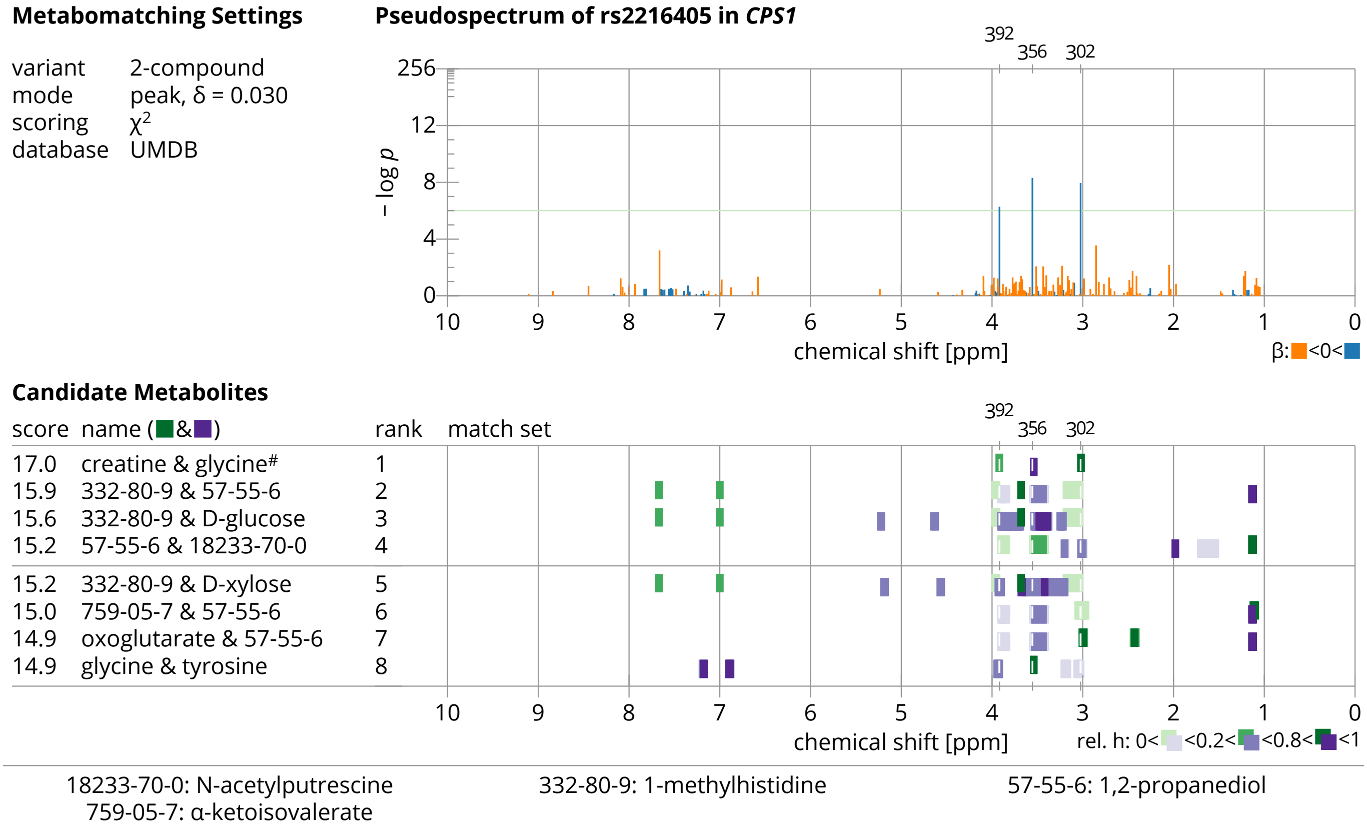

(C) SHIP, *CPS1*. Peak-mode, χ^2-scoring 2-compound metabomatching. The rs2216405 pseudospectrum is characterized by three significantly associated features. 2-compound metabomatching ranks the glycine & creatine pair first, with glycine a one-to-one match with feature 3.56, and creatine a strong two-to-two match with features 3.02 and 3.92. Metabomatching in multiplet mode gives significantly different results (see Fig S2E, *CPS1*/M/2c)

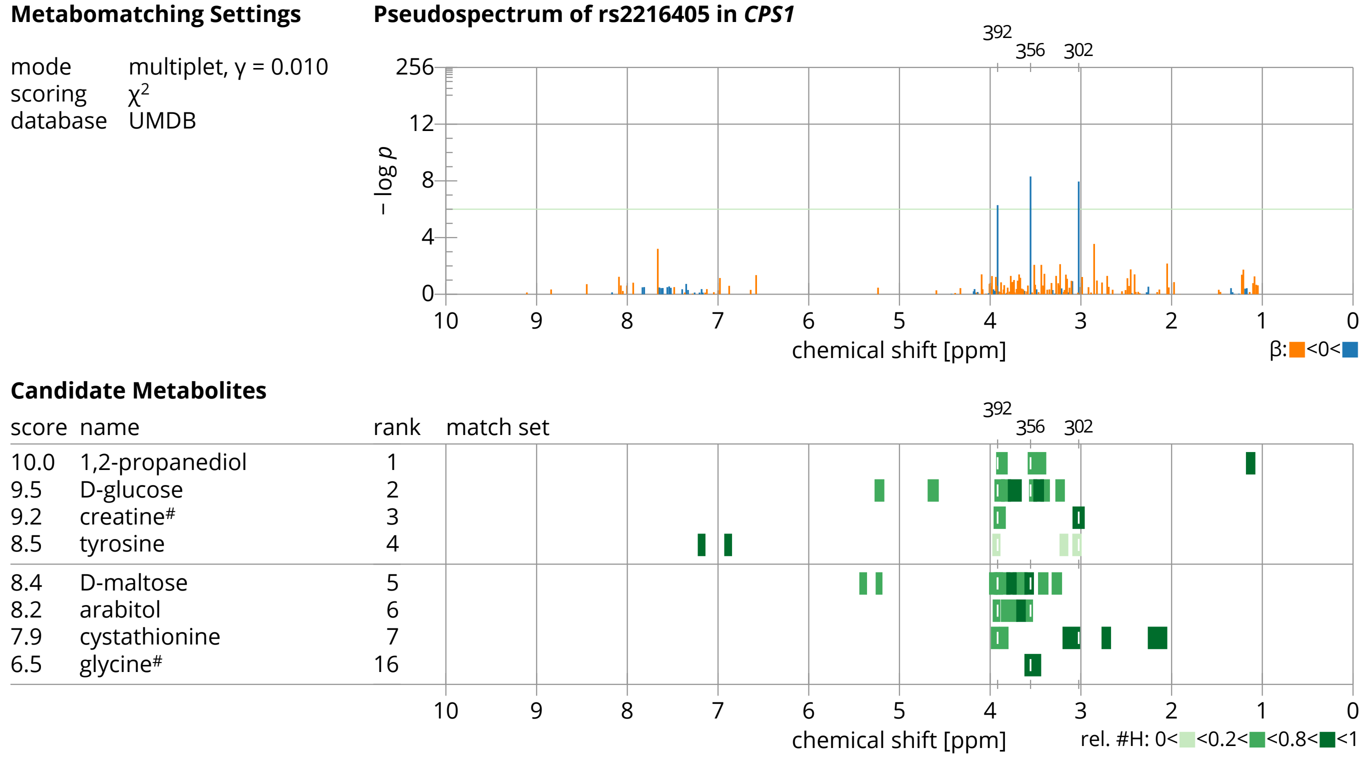

(D) SHIP, *CPS1*. Multiplet-mode, χ^2-scoring metabomatching. The rs2216405 pseudospectrum is characterized by three significantly associated features. While these features do correspond to creatine and glycine, the multiplet description of the metabolites are characterized by wide multiplet ranges. As a result, the ranks of creatine and glycine are significantly lower than in peak-mode metabomatching (see Fig S2B, *CPS1*). Given the presence of two reference metabolites, we run 2-compound metabomatching (see Fig S2C, *CPS1*/2c)

Navigation:

CoLaus	*SLC6A20*	*AGXT2*	*SOSTDC1*	*PYROXD2*	*SLC6A13*	*HPD*	*HPD*/M	*PNMT*	*SLC7A9*	*UPS9*
SHIP	*DAB1*	*CPS1*	*CPS1*/2c	*CPS1*/M	*CPS1*/M/2c	*XYLB*	*XYLB*/M	*SLC6A20*	*ENTPPL*	*SLC6A19*	*AGXT2*
	DMGDH	*SLC36A2*	*NAT2*	*NAT2*/±	*SLC6A13*	*HPD*	*HPD*/2c	*HPD*/M	*HPD*/M/2c	*PNMT*	*SLC7A9*

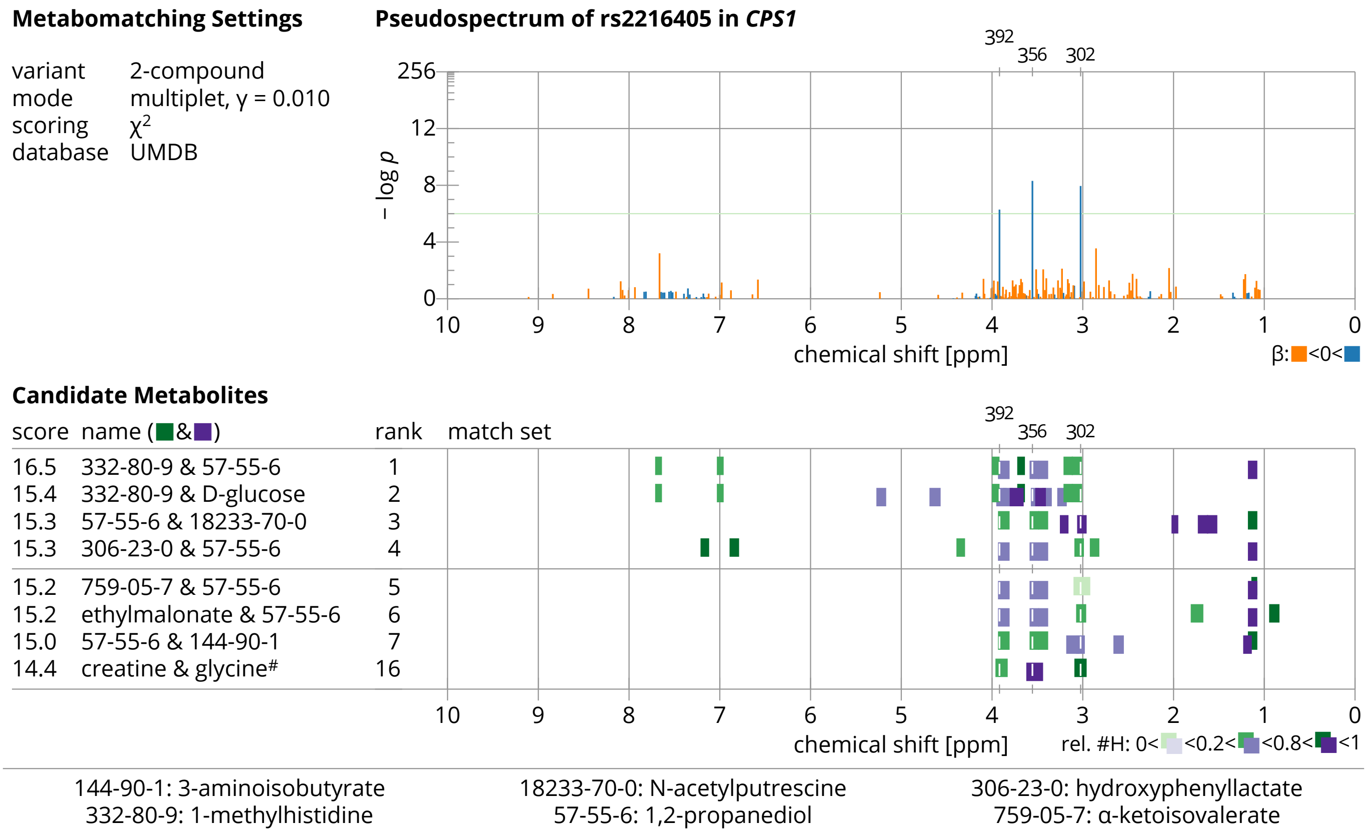

(E) SHIP, *CPS1*. Multiplet-mode, χ^2-scoring 2-compound metabomatching. The rs2216405 pseudospectrum is characterized by three significantly associated features. While these features do correspond to creatine and glycine, the multiplet description of the metabolites are characterized by wide multiplet ranges. As a result, the rank of the pair is significantly lower than in 2-compound peak-mode metabomatching (see Fig S2C, *CPS1*/2c).

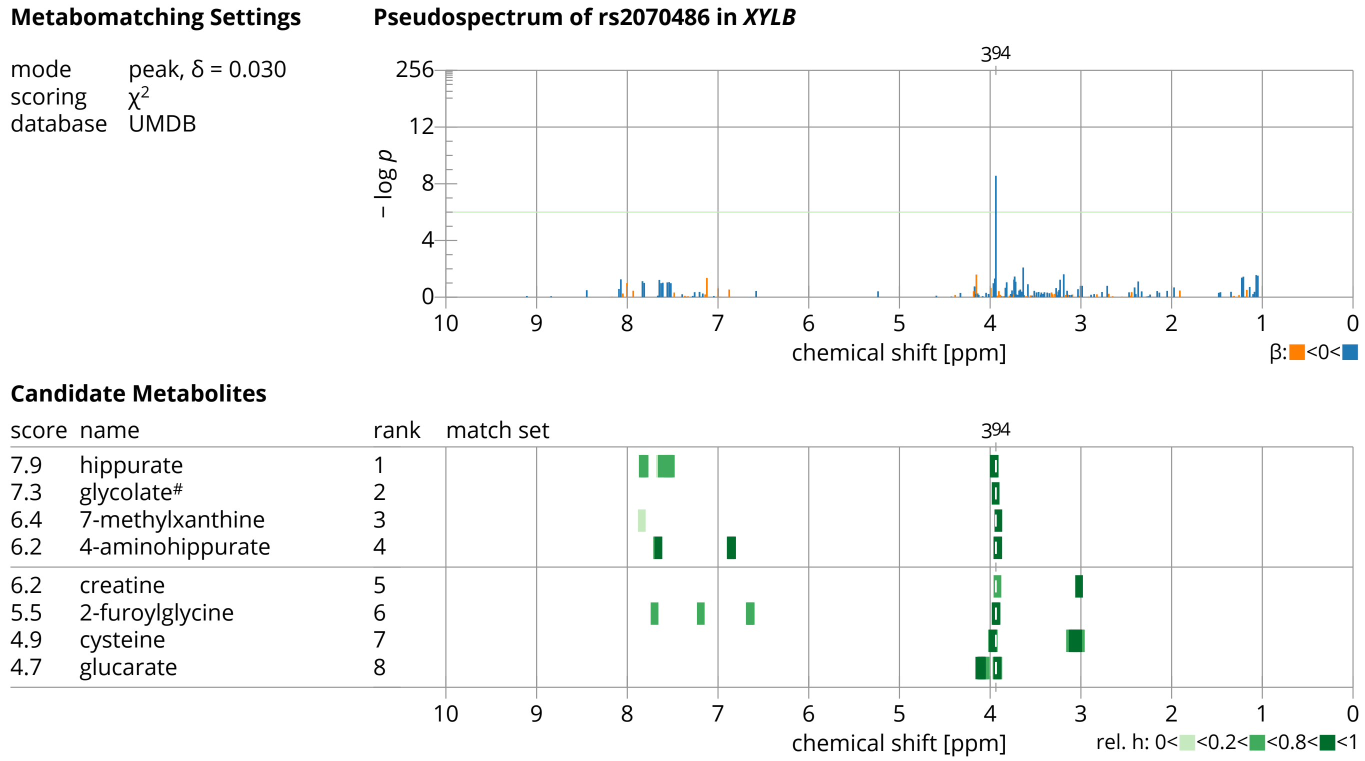

(F) SHIP, *XYLB*. Peak-mode, χ^2-scoring metabomatching. The rs2070486 pseudospectrum is characterized by a single significantly associated feature: feature 3.94. Feature 3.94 corresponds to the single peak NMR spectrum of glycolate. Producing only a single peak match, however, glycolate does not notably outscore other metabolites that match feature 3.94. Metabomatching in multiplet mode gives significantly different results (see Fig S2G, *XYLB*/M)

Navigation:

CoLaus	*SLC6A20*	*AGXT2*	*SOSTDC1*	*PYROXD2*	*SLC6A13*	*HPD*	*HPD/M*	*PNMT*	*SLC7A9*	*UPS9*	
SHIP	*DAB1*	*CPS1*	*CPS1/2c*	*CPS1/M*	*CPS1/M/2c*	*XYLB*	*XYLB/M*	*SLC6A20*	*ENTPPL*	*SLC6A19*	*AGXT2*
	DMGDH	*SLC36A2*	*NAT2*	*NAT2/±*	*SLC6A13*	*HPD*	*HPD/2c*	*HPD/M*	*HPD/M/2c*	*PNMT*	*SLC7A9*

Metabomatching Settings

mode	multiplet, γ = 0.010
scoring	χ^2
database	UMDB

Pseudospectrum of rs2070486 in *XYLB*

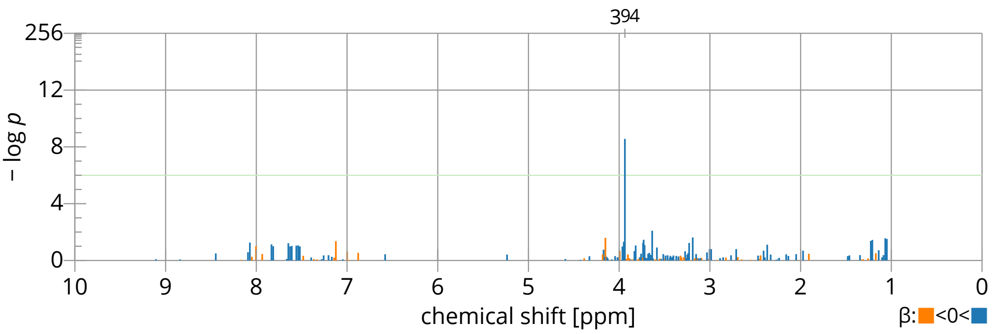

Candidate Metabolites

score	name	rank
8.6	hippurate	1
6.1	4-aminohippurate	2
5.5	2-furoylglycine	3
5.2	cysteine	4
5.0	glucarate	5
4.8	arabitol	6
4.8	serine	7
3.7	glycolate#	16

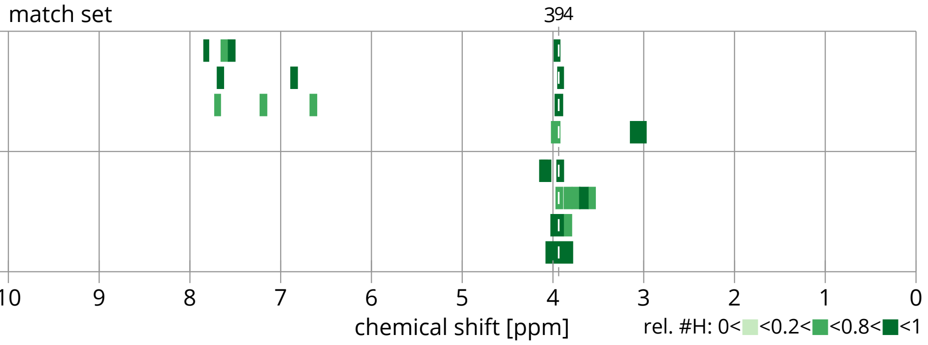

(G) SHIP, *XYLB*. Multiplet-mode, χ^2-scoring metabomatching. The rs2070486 pseudospectrum is characterized by a single significantly associated feature: feature 3.94. While the single multiplet range of the glycolate spectrum matches feature 3.94, it is defined by a wide range. This lowers its match score, and allows other metabolites with multiplets matching 3.94 to outrank it.

Metabomatching Settings

mode	peak, δ = 0.030
scoring	χ^2
database	UMDB

Pseudospectrum of rs17279437 in *SLC6A20*

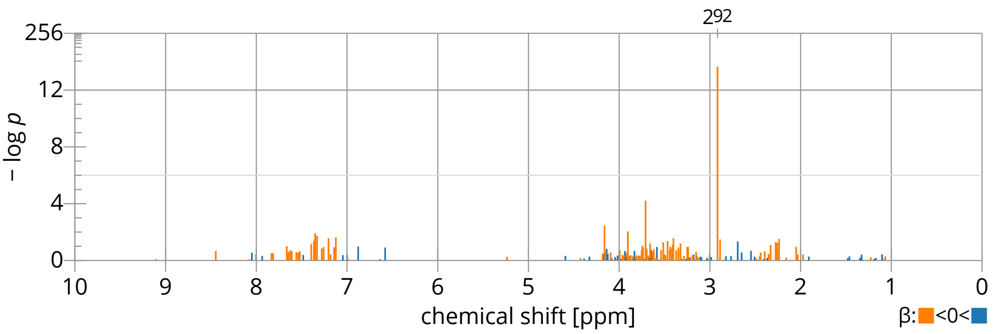

Candidate Metabolites

score	name	rank
21.2	dimethylglycine#	1
21.1	trimethylamine	2
19.5	N-methylhydantoin	3
17.8	2-methyl-3-ketovalerate	4
16.7	3-methyl-2-oxovalerate	5
16.2	hydroxyphenyllactate	6
16.2	asparagine	7
14.5	3-aminoisobutyrate	8

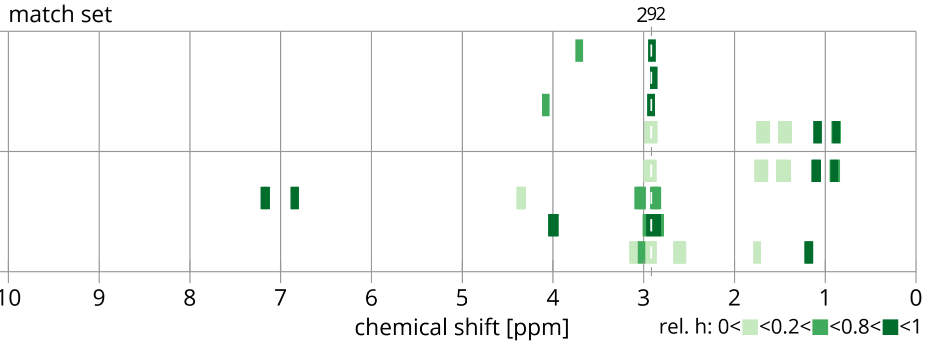

(H) SHIP, *SLC6A20*. Peak-mode, χ^2-scoring metabomatching. The rs17279437 pseudospectrum is characterized by a single significantly associated feature, feature 2.92, with a secondary signal at 3.71. The dimethylglycine spectrum matches both feature 2.92, and the secondary signal.

Navigation:

CoLaus	*SLC6A20*	*AGXT2*	*SOSTDC1*	*PYROXD2*	*SLC6A13*	*HPD*	*HPD/M*	*PNMT*	*SLC7A9*	*UPS9*	
SHIP	*DAB1*	*CPS1*	*CPS1/2c*	*CPS1/M*	*CPS1/M/2c*	*XYLB*	*XYLB/M*	*SLC6A20*	*ENTPPL*	*SLC6A19*	*AGXT2*
	DMGDH	*SLC36A2*	*NAT2*	*NAT2/±*	*SLC6A13*	*HPD*	*HPD/2c*	*HPD/M*	*HPD/M/2c*	*PNMT*	*SLC7A9*

Metabomatching Settings

mode	peak, δ = 0.030
scoring	χ^2
database	UMDB

Pseudospectrum of rs7654111 in *ENTPPL*

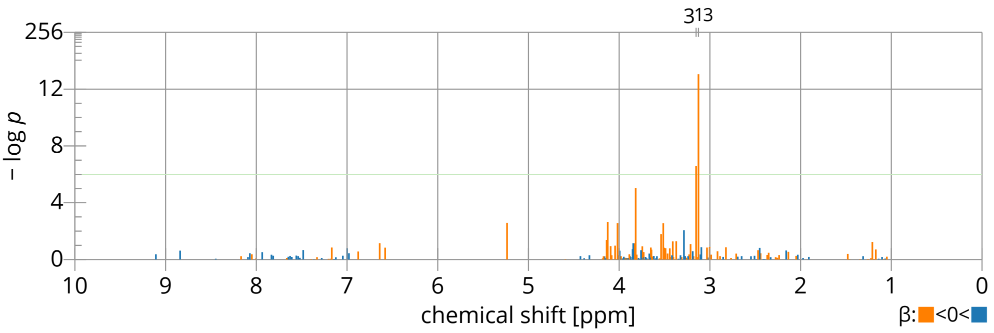

Candidate Metabolites

score	name	rank
19.8	ethanolamine#	1
18.6	dimethyl-sulfone	2
17.0	dihydrothymine	3
16.5	methylmalonate	4
14.9	histidine	5
14.2	malonate	6
13.7	phenylalanine	7
13.6	cystathionine	8

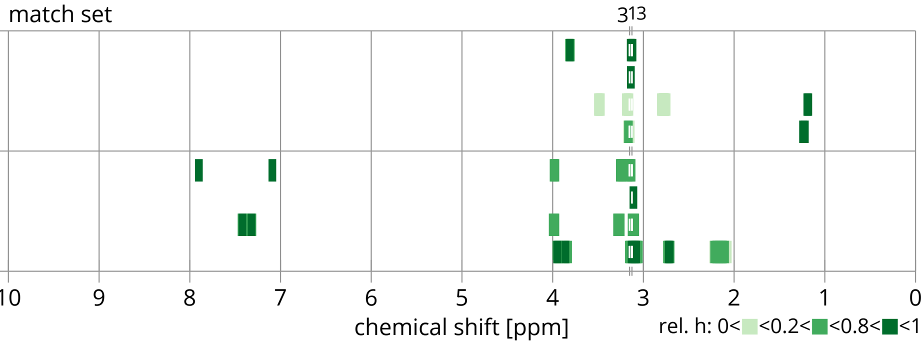

(I) SHIP, *ENTPPL*. Peak-mode, χ^2-scoring metabomatching. The rs7654111 pseudospectrum is characterized by a significantly associated feature cluster, led by feature 3.13, with a secondary signal at 3.81. The ethanolamine spectrum matches both feature 3.13, and the secondary signal.

Metabomatching Settings

mode	peak, δ = 0.030
scoring	χ^2
database	UMDB

Pseudospectrum of rs7719875 in *SLC6A19*

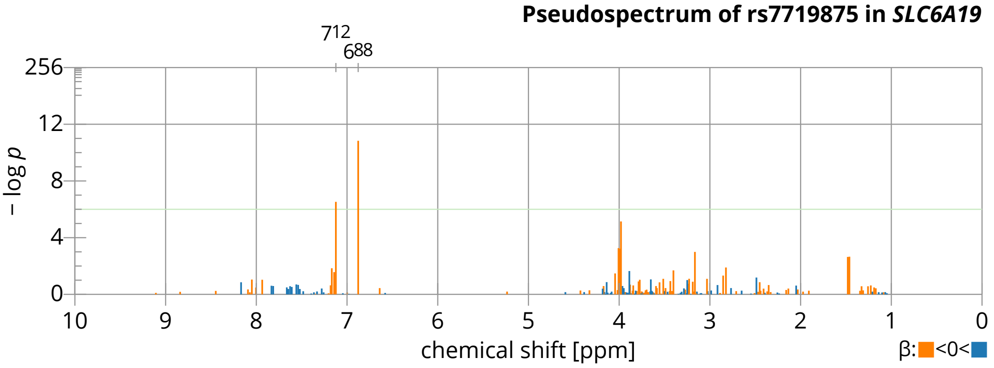

Candidate Metabolites

score	name	rank
14.4	p-hydroxyphenylacetate	1
12.8	ferulate	2
10.9	pyrocatechol	3
9.8	4-hydroxybenzoate	4
9.7	asparagine	5
9.7	2-hydroxybutyrate	6
8.2	tyrosine#	8
8.2	histidine#	9

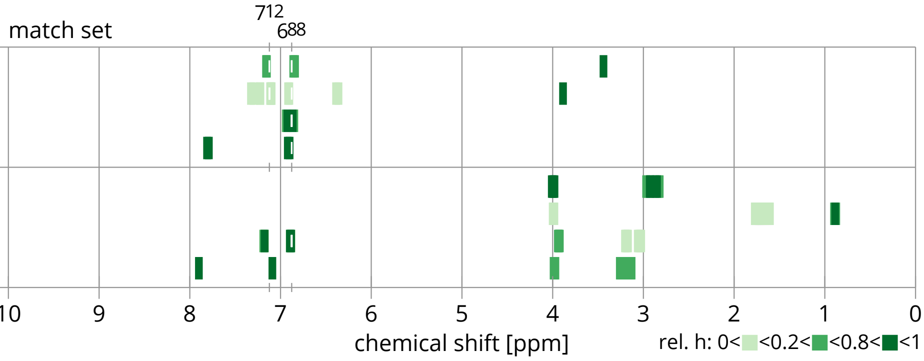

(J) SHIP, *SLC6A19*. Peak-mode, χ^2-scoring metabomatching. The rs7719875 pseudospectrum is characterized by two significantly associated features (feature 7.12 and feature 6.88), with a secondary cluster at 4.00. The spectrum of control metabolite tyrosine matches feature 6.88, but also contains other, non matching, peak clusters. Feature 7.12 is located at 7.123 precisely, narrowly missing the match subsets of both control metabolites ([7.060,7.120] for histidine, and [7.140,7.223] for tyrosine). The histidine spectrum obtains a comparatively high score by matching the secondary pseudospectrum cluster at 4.00.

Navigation:

CoLaus	*SLC6A20*	*AGXT2*	*SOSTDC1*	*PYROXD2*	*SLC6A13*	*HPD*	*HPD/M*	*PNMT*	*SLC7A9*	*UPS9*	
SHIP	*DAB1*	*CPS1*	*CPS1/2c*	*CPS1/M*	*CPS1/M/2c*	*XYLB*	*XYLB/M*	*SLC6A20*	*ENTPPL*	*SLC6A19*	*AGXT2*
	DMGDH	*SLC36A2*	*NAT2*	*NAT2/±*	*SLC6A13*	*HPD*	*HPD/2c*	*HPD/M*	*HPD/M/2c*	*PNMT*	*SLC7A9*

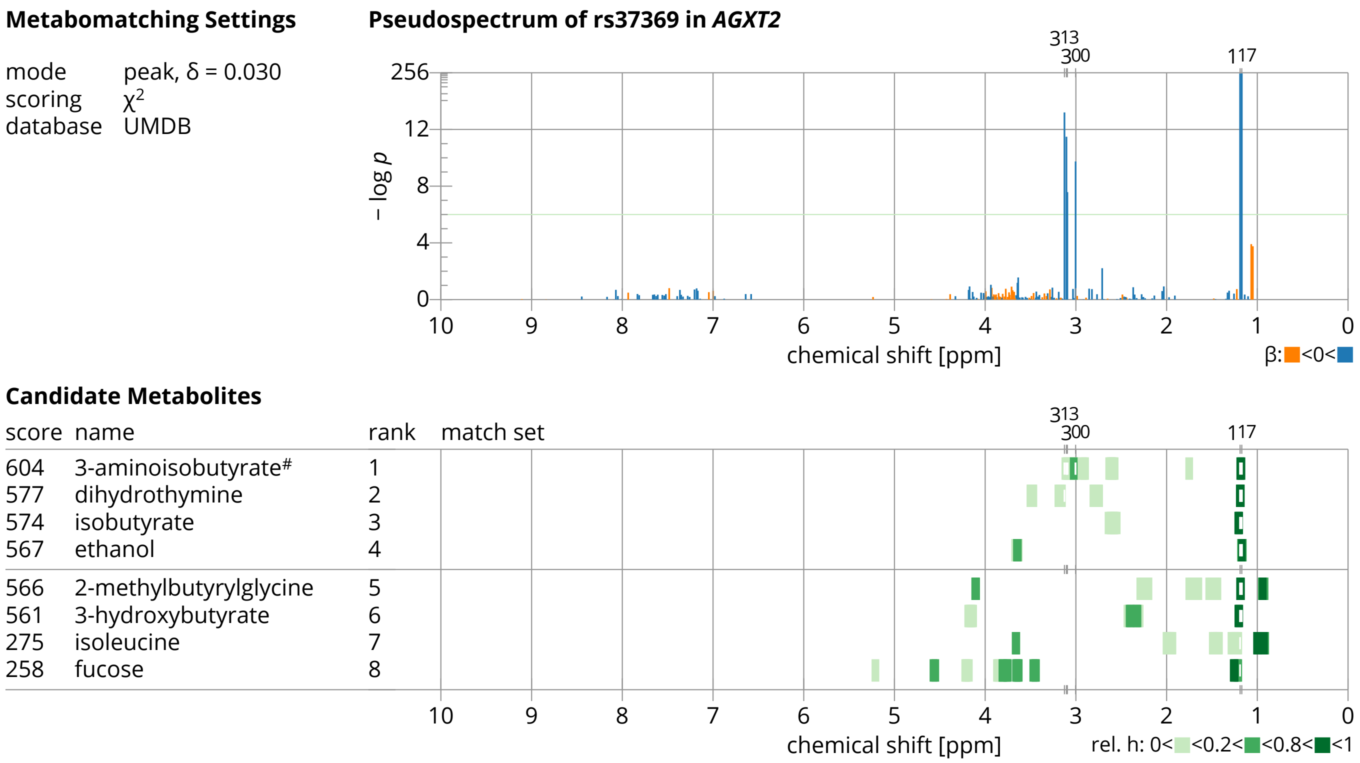

(K) SHIP, *AGXT2*. Peak-mode, χ^2-scoring metabomatching. The rs37369 pseudospectrum is characterized by three significantly associated feature clusters, with equal effect directions. The 3-aminoisobutyrate spectrum produces a strong match, with three peak clusters matching all three significantly associated feature clusters.

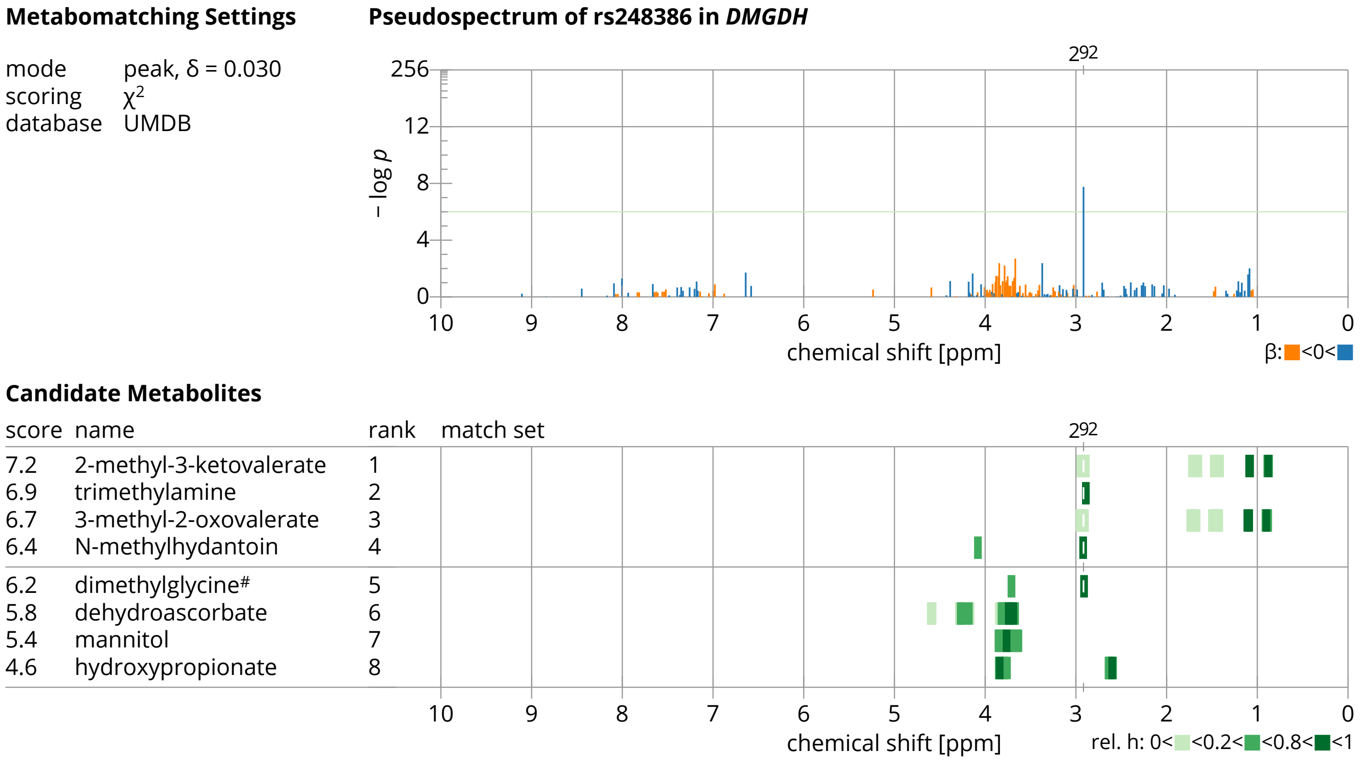

(L) SHIP, *DMGDH*. Peak-mode, χ^2-scoring metabomatching. The rs248386 pseudospectrum is characterized by a single significantly associated feature: feature 2.92. The dimethylglycine spectrum matches feature 2.92, but its second cluster is unmatched. Compare with *SLC6A20*, for which dimethylglycine is also the reference metabolite (Fig S2H).

Navigation:

CoLaus	*SLC6A20*	*AGXT2*	*SOSTDC1*	*PYROXD2*	*SLC6A13*	*HPD*	*HPD/M*	*PNMT*	*SLC7A9*	*UPS9*
SHIP	*DAB1*	*CPS1*	*CPS1/2c*	*CPS1/M*	*CPS1/M/2c*	*XYLB*	*XYLB/M*	*SLC6A20*	*ENTPPL*	*SLC6A19*	*AGXT2*
	DMGDH	*SLC36A2*	*NAT2*	*NAT2/±*	*SLC6A13*	*HPD*	*HPD/2c*	*HPD/M*	*HPD/M/2c*	*PNMT*	*SLC7A9*

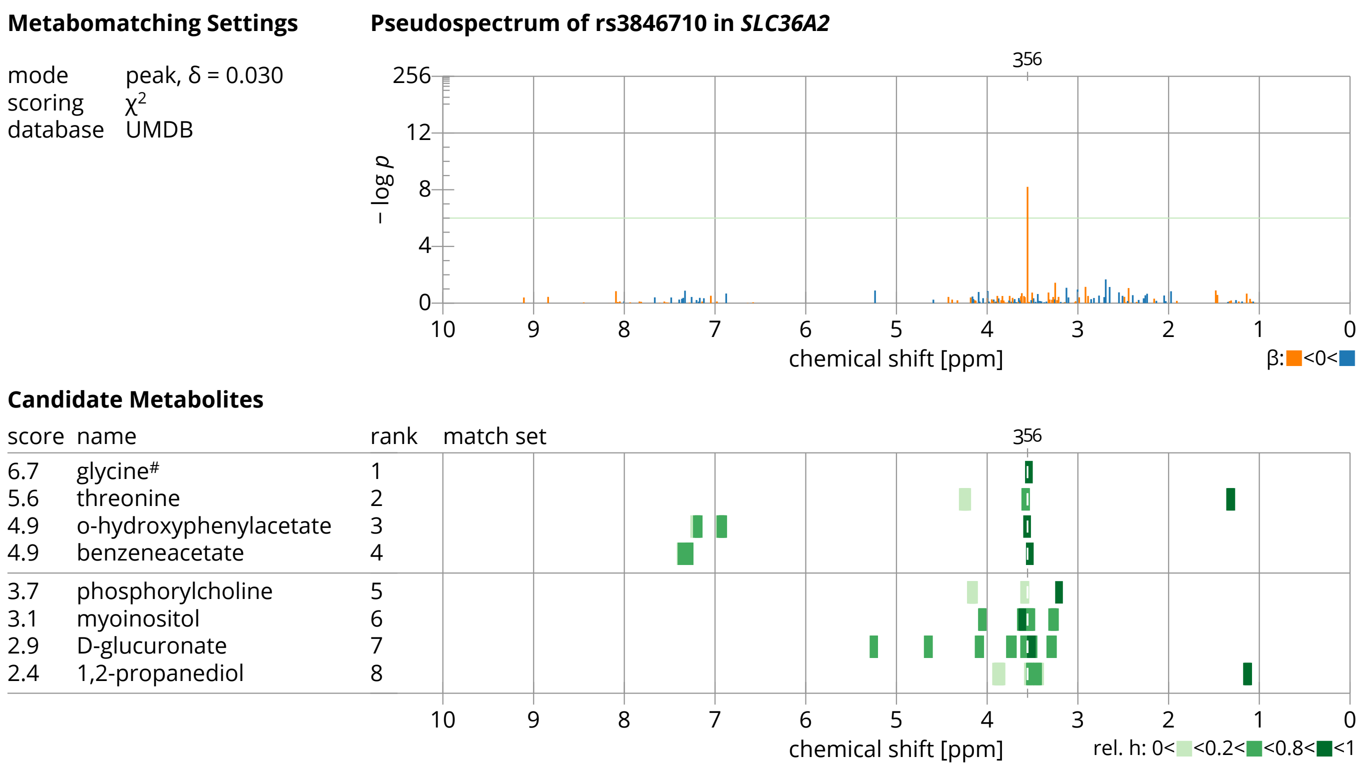

(M) SHIP, *SLC36A2*. Peak-mode, χ^2-scoring metabomatching. The rs3846710 pseudospectrum is characterized by a single significantly associated feature: feature 3.56. Feature 3.56 is matched by the single peak of the glycine spectrum. Producing only a single peak match, however, glycine does not notably outscore other metabolites that match feature 3.56.

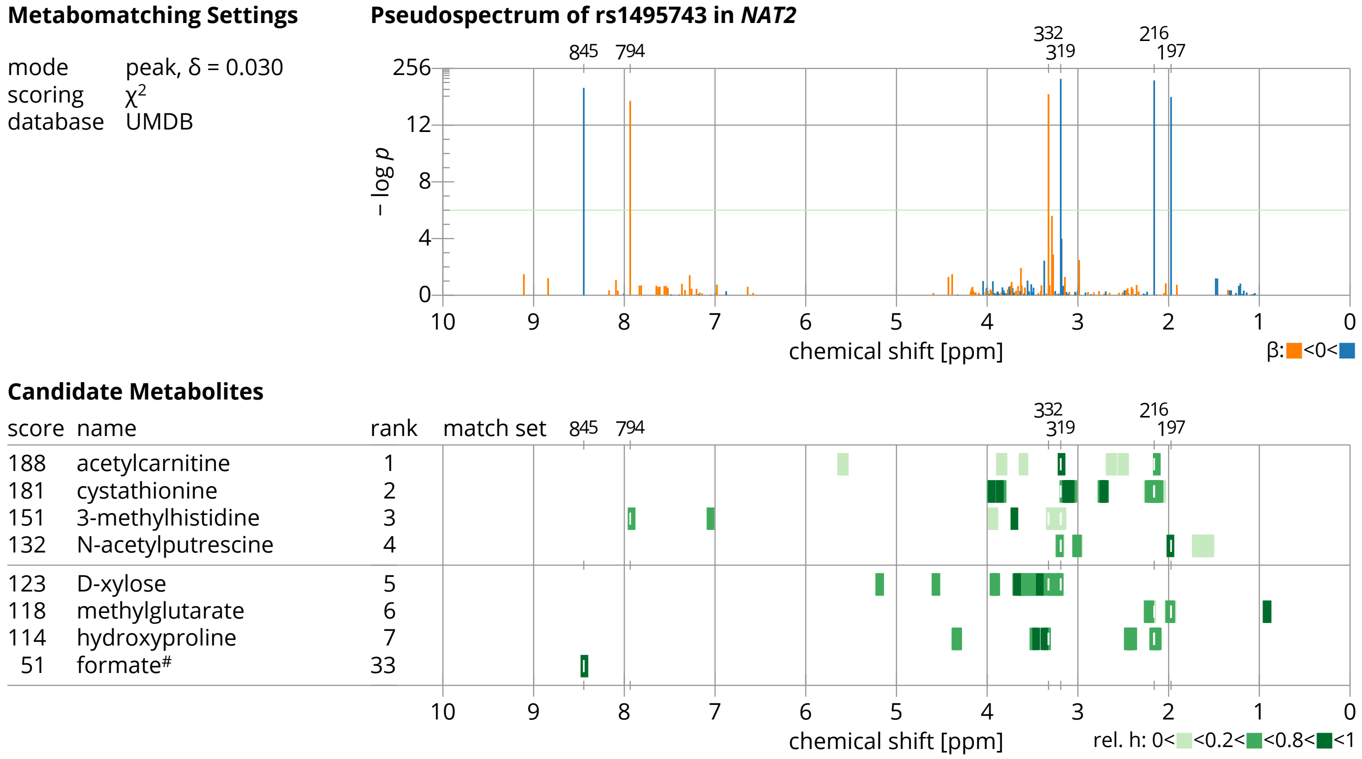

(N) SHIP, *NAT2*. Peak-mode, χ^2-scoring metabomatching. The rs1495743 pseudospectrum is characterized by six significantly associated features: four with positive, two with negative effect sizes. The single peak of formate matches feature 8.45, but this is not sufficient to outrank metabolites matching other features. The pseudospectrum suggests at least three underlying metabolites, of which two with positive, and one with negative effect size. We therefore apply ±-2-compound metabomatching (Fig S2O, *NAT2/±*).

Navigation:

CoLaus	*SLC6A20*	*AGXT2*	*SOSTDC1*	*PYROXD2*	*SLC6A13*	*HPD*	*HPD/M*	*PNMT*	*SLC7A9*	*UPS9*
SHIP	*DAB1*	*CPS1*	*CPS1/2c*	*CPS1/M*	*CPS1/M/2c*	*XYLB*	*XYLB/M*	*SLC6A20*	*ENTPPL*	*SLC6A19*	*AGXT2*
	DMGDH	*SLC36A2*	*NAT2*	*NAT2/±*	*SLC6A13*	*HPD*	*HPD/2c*	*HPD/M*	*HPD/M/2c*	*PNMT*	*SLC7A9*

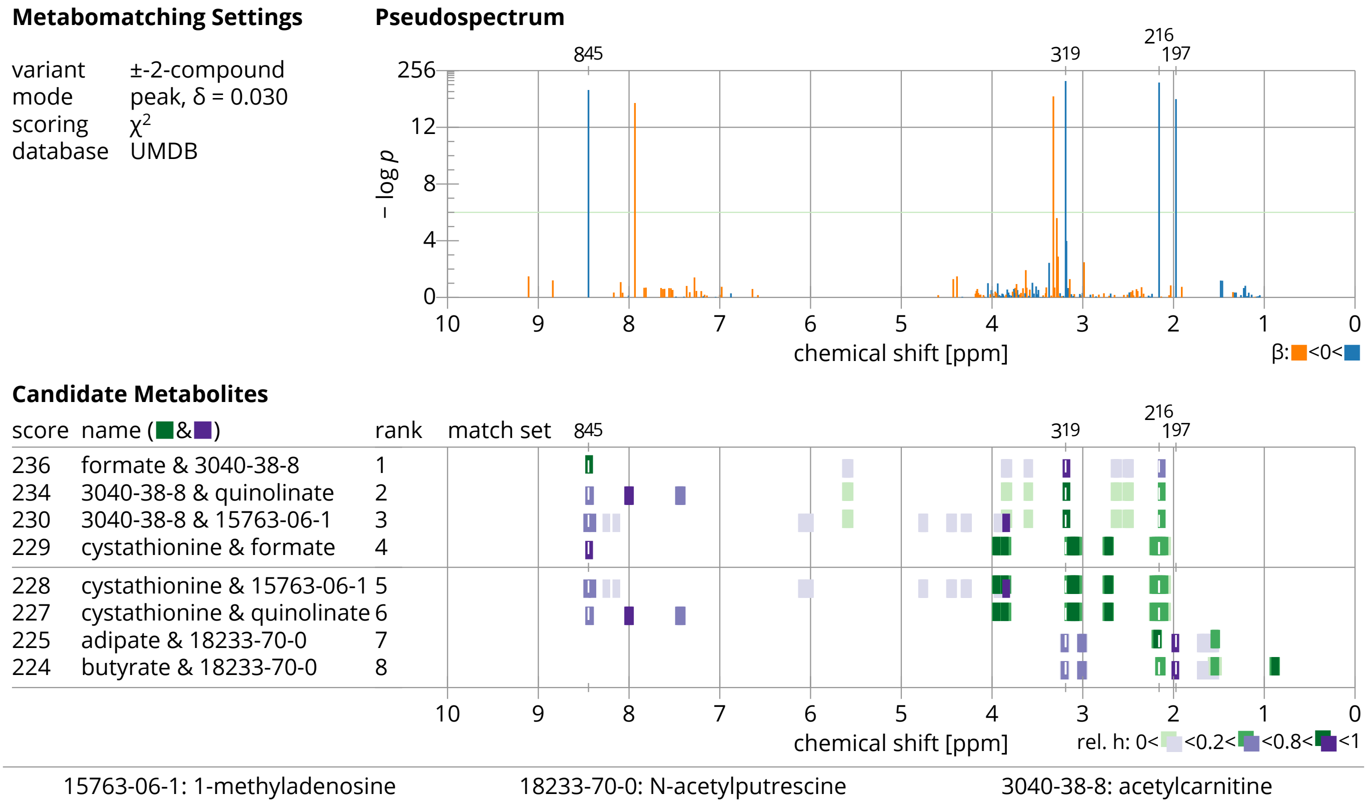

(O) SHIP, *NAT2*. Peak-mode, χ^2-scoring ±-compound metabomatching. The rs1495743 pseudospectrum is characterized by six significantly associated features: four with positive, two with negative effect sizes. ±-2-compound metabomatching, ranks a metabolite pair which includes formate first.

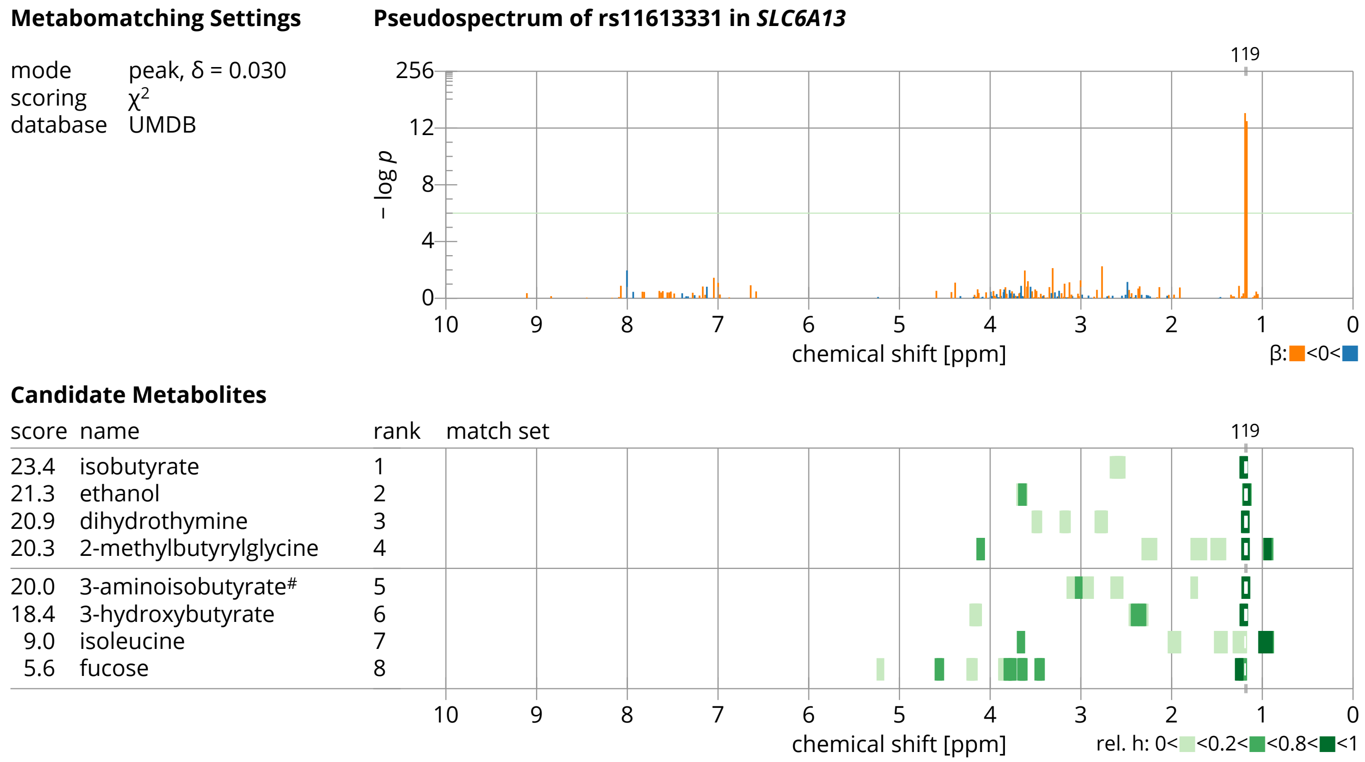

(P) SHIP, *SLC6A13*. Peak-mode, χ^2-scoring metabomatching. The rs11613331 pseudospectrum is characterized by a cluster of significantly associated features, led by feature 1.19. One of the main peaks of the 3-aminoisobutyrate spectrum is matched by feature 1.19, but all others peaks are unmatched in the pseudospectrum. The five other metabolites matching feature 1.19 attain similar scores, with isobutyrate ranking first because it has the fewest non-matched peaks. Compare to the far better match of 3-aminoisobutyrate with the *AGXT2* SNP pseudospectrum (Fig S2K).

Navigation:

CoLaus	*SLC6A20*	*AGXT2*	*SOSTDC1*	*PYROXD2*	*SLC6A13*	*HPD*	*HPD/M*	*PNMT*	*SLC7A9*	*UPS9*
SHIP	*DAB1*	*CPS1*	*CPS1/2c*	*CPS1/M*	*CPS1/M/2c*	*XYLB*	*XYLB/M*	*SLC6A20*	*ENTPPL*	*SLC6A19*	*AGXT2*
	DMGDH	*SLC36A2*	*NAT2*	*NAT2/±*	*SLC6A13*	*HPD*	*HPD/2c*	*HPD/M*	*HPD/M/2c*	*PNMT*	*SLC7A9*

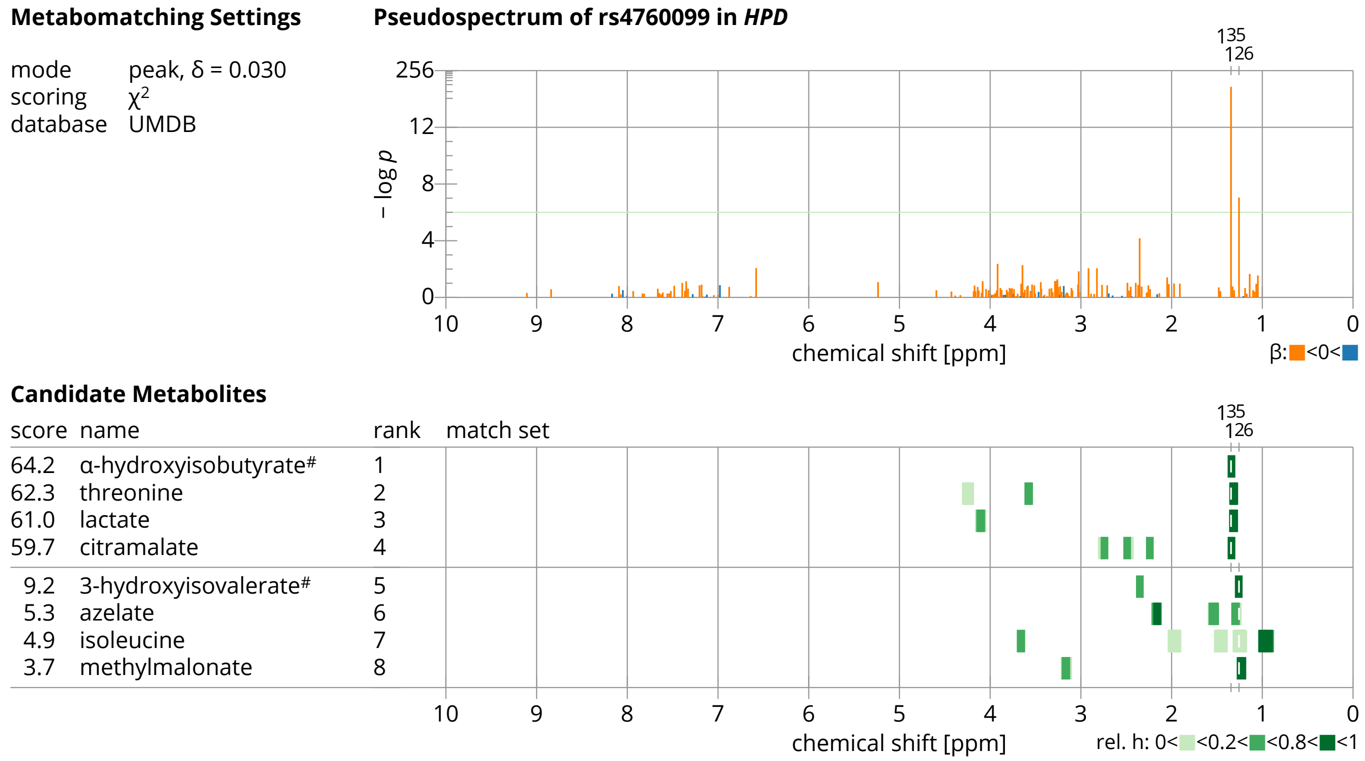

(Q) SHIP, *HPD*. Peak-mode, χ^2-scoring metabomatching. The rs4760099 pseudospectrum is characterized by two significantly associated features: feature 1.26 and feature 1.35. Feature 1.35 matches the single peak spectrum of α-hydroxyisobutyrate. Feature 1.26 matches the main peak of spectrum of 3-hydroxyisovalerate. In contrast to the similar pseudospectrum in CoLaus (Fig S1F and 1G), the significantly associated features are well aligned with their corresponding metabolite spectra. Given the presence of two reference metabolites, we run 2-compound metabomatching (see Fig S2R, *HPD*/2c)

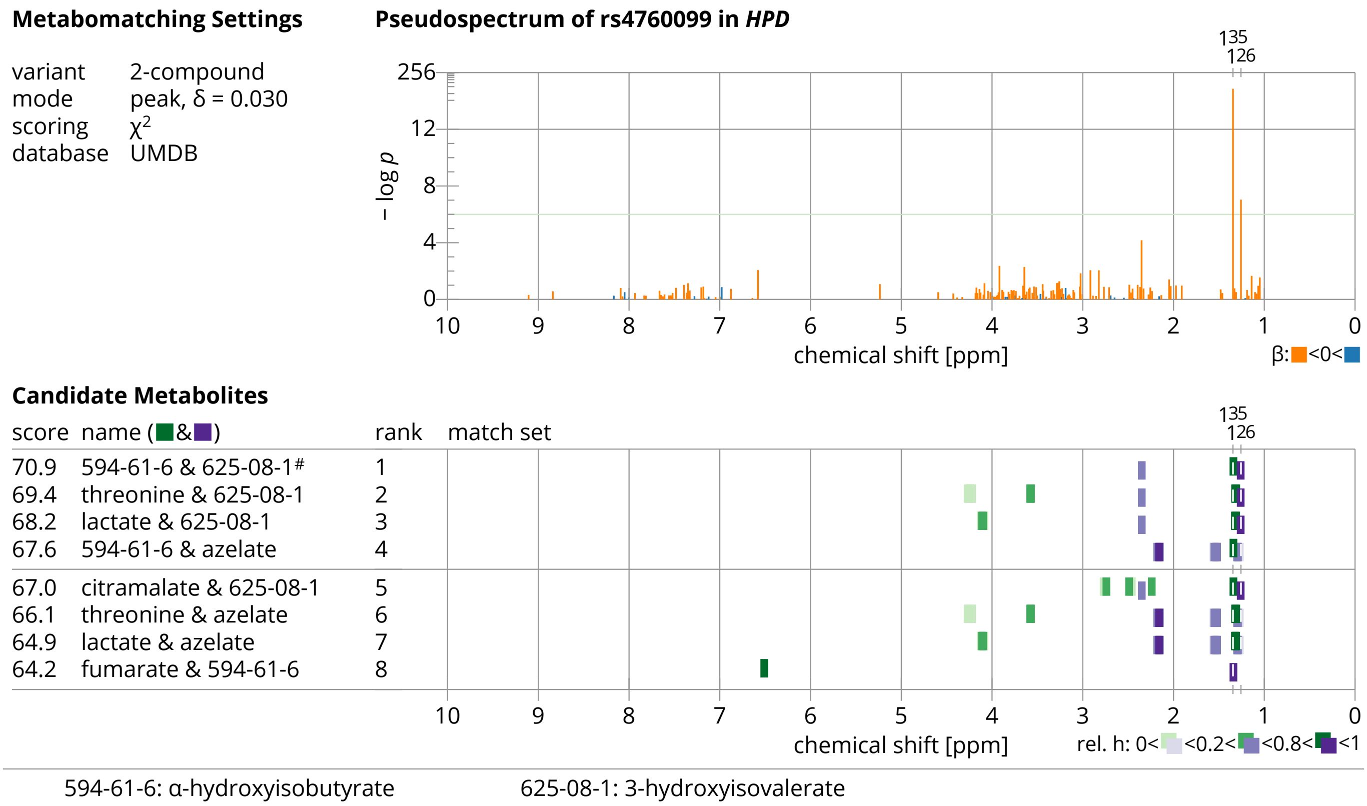

(R) SHIP, *HPD*. Peak-mode, χ^2-scoring 2-compound metabomatching. The rs4760099 pseudospectrum is characterized by a two significantly associated features: feature 1.26 and feature 1.35. 2-compound metabomatching ranks the α-hydroxyisobutyrate & 3-hydroxyisovalerate pair first.

Navigation:

CoLaus	*SLC6A20*	*AGXT2*	*SOSTDC1*	*PYROXD2*	*SLC6A13*	*HPD*	*HPD/M*	*PNMT*	*SLC7A9*	*UPS9*	
SHIP	*DAB1*	*CPS1*	*CPS1/2c*	*CPS1/M*	*CPS1/M/2c*	*XYLB*	*XYLB/M*	*SLC6A20*	*ENTPPL*	*SLC6A19*	*AGXT2*
	DMGDH	*SLC36A2*	*NAT2*	*NAT2/±*	*SLC6A13*	*HPD*	*HPD/2c*	*HPD/M*	*HPD/M/2c*	*PNMT*	*SLC7A9*

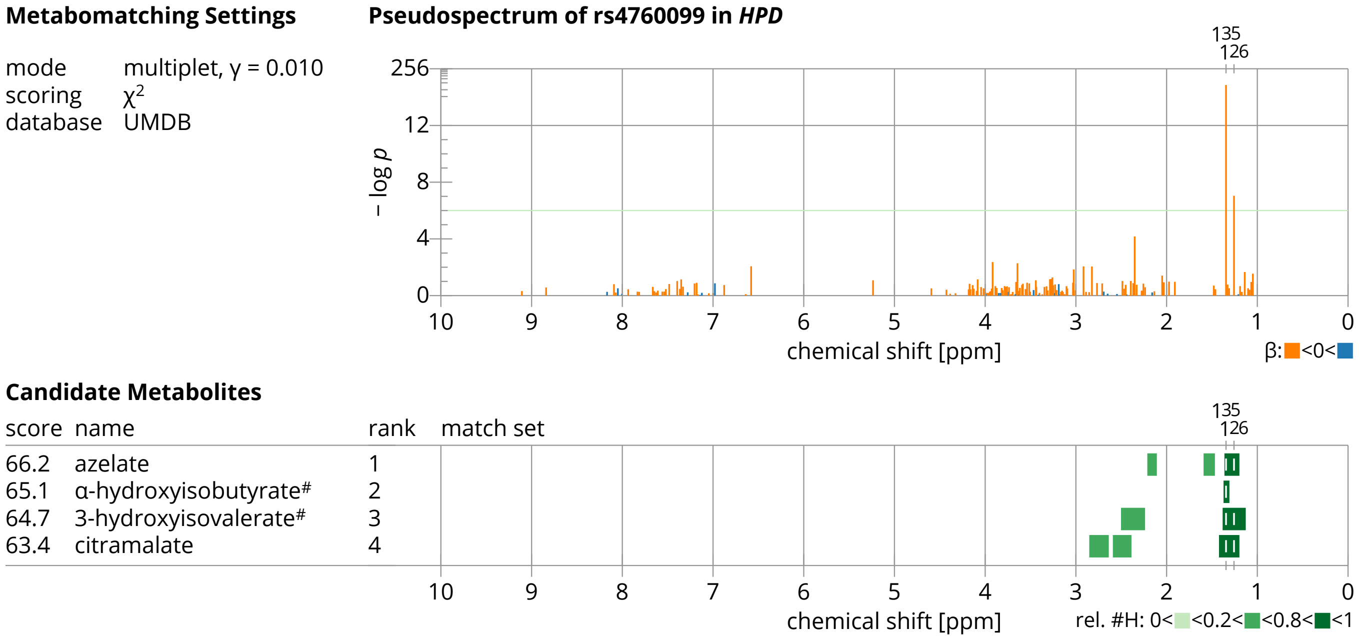

(S) SHIP, *HPD*. Multiplet-mode, χ^2-scoring metabomatching. The rs4760099 pseudospectrum is characterized by two significantly associated features: feature 1.26 and feature 1.35. Feature 1.35 produces a one-to-one match with the spectrum of control metabolite α-hydroxyisobutyrate. For 3-hydroxyisovalerate, the multiplet range that encloses the spectrum peak 1.26 (see Fig S2Q), now enclose both feature 1.26 and feature 1.35. This artifact results in a higher, though undeserved, rank for 3-hydroxyisovalerate. Given the presence of two reference metabolites, we run 2-compound metabomatching (see Fig S2T, *HPD/2c*)

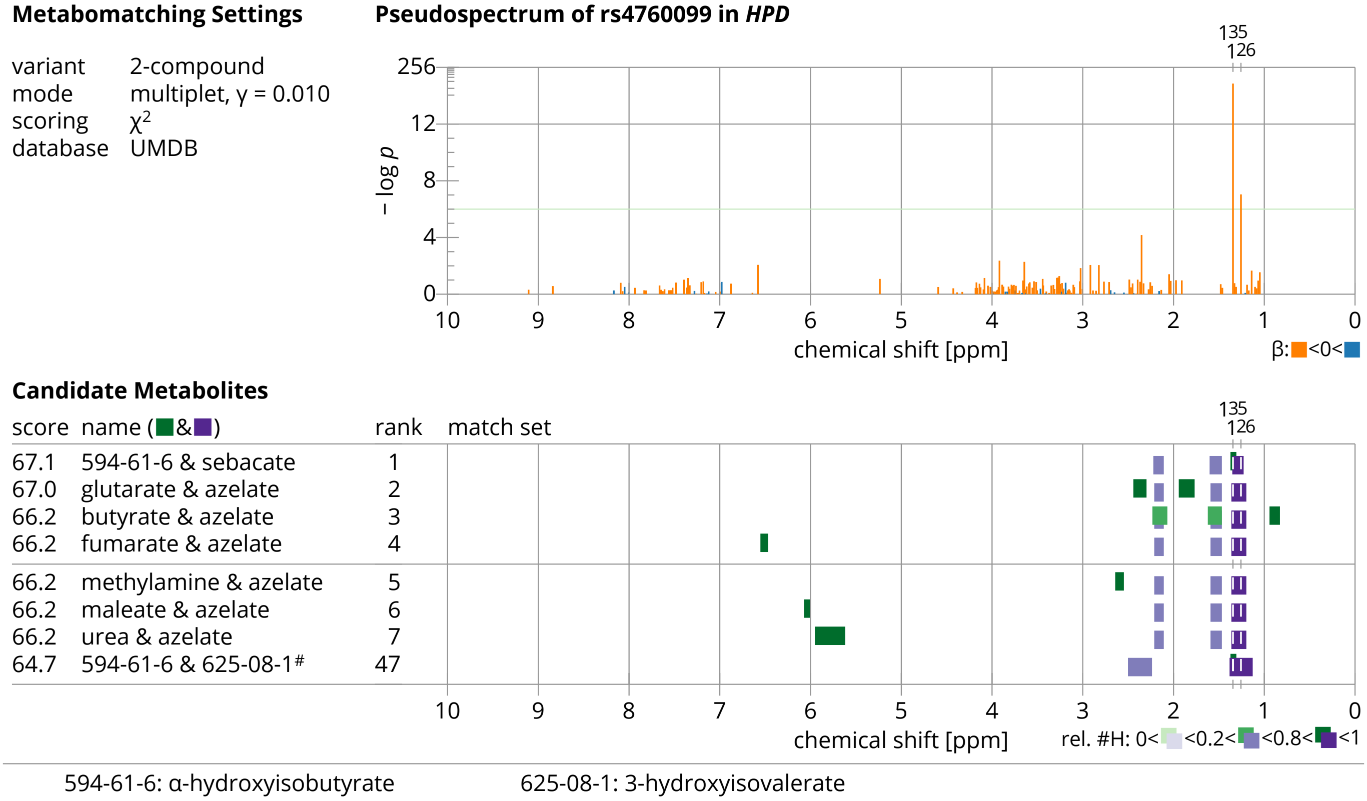

(T) SHIP, *HPD*. Multiplet-mode, χ^2-scoring 2-compound metabomatching. The rs4760099 pseudospectrum is characterized by a two significantly associated features: feature 1.26 and feature 1.35. Wide multipet ranges in the multiplet descriptions of 3-hydroxyvalerate and competing metabolite azelate invert the rankings seen in peak-mode metabomatching (see Fig S2R). 3-hydroxyisovalerate and azelate both match feature 1.26 and feature 1.35. Because the azelate match set contains fewer additional features, azelate pairs outrank 3-hydroxyisovalerate pairs. The match set for α-hydroxyisobutyrate is a subset of the match sets of both azelate and 3-hydroxyisovalerate, and does not contribute to the score.

Navigation:

CoLaus	*SLC6A20*	*AGXT2*	*SOSTDC1*	*PYROXD2*	*SLC6A13*	*HPD*	*HPD/M*	*PNMT*	*SLC7A9*	*UPS9*	
SHIP	*DAB1*	*CPS1*	*CPS1/2c*	*CPS1/M*	*CPS1/M/2c*	*XYLB*	*XYLB/M*	*SLC6A20*	*ENTPPL*	*SLC6A19*	*AGXT2*
	DMGDH	*SLC36A2*	*NAT2*	*NAT2/±*	*SLC6A13*	*HPD*	*HPD/2c*	*HPD/M*	*HPD/M/2c*	*PNMT*	*SLC7A9*

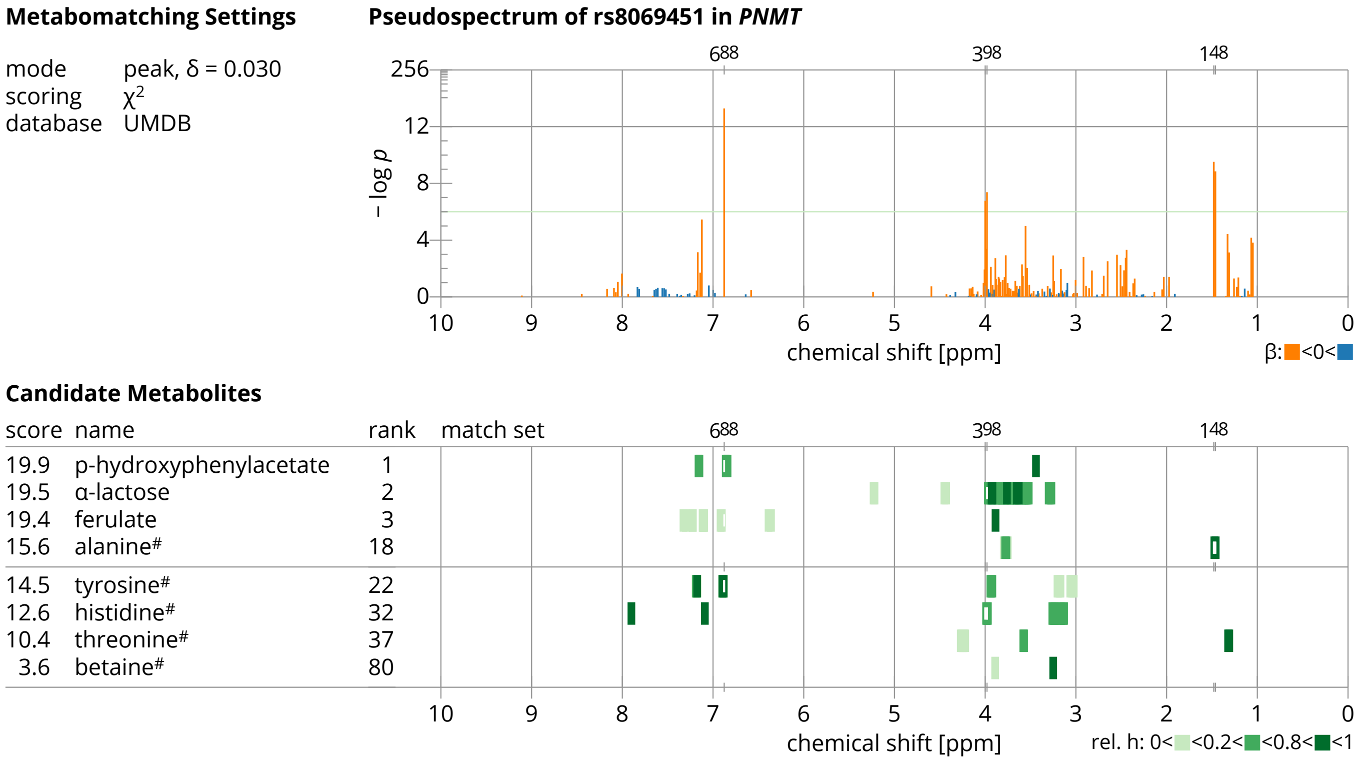

(U) SHIP, *PNMT*. Peak-mode, χ^2-scoring metabomatching. The rs8069451 pseudospectrum is characterized by three clusters of significantly associated features: feature 1.48, feature 3.98, and feature 6.88, as well as a number of secondary signals. In the targeted mGWAS, rs8069451 was found to be associated with five metabolites: alanine, betaine, histidine, threonine, and tyrosine. Of these, the spectra of alanine, tyrosine, and histidine each match one of the significantly associated features, and are therefore testable. Because their spectra contain many additional, unmatched, peaks, however, none obtain high metabomatching scores.

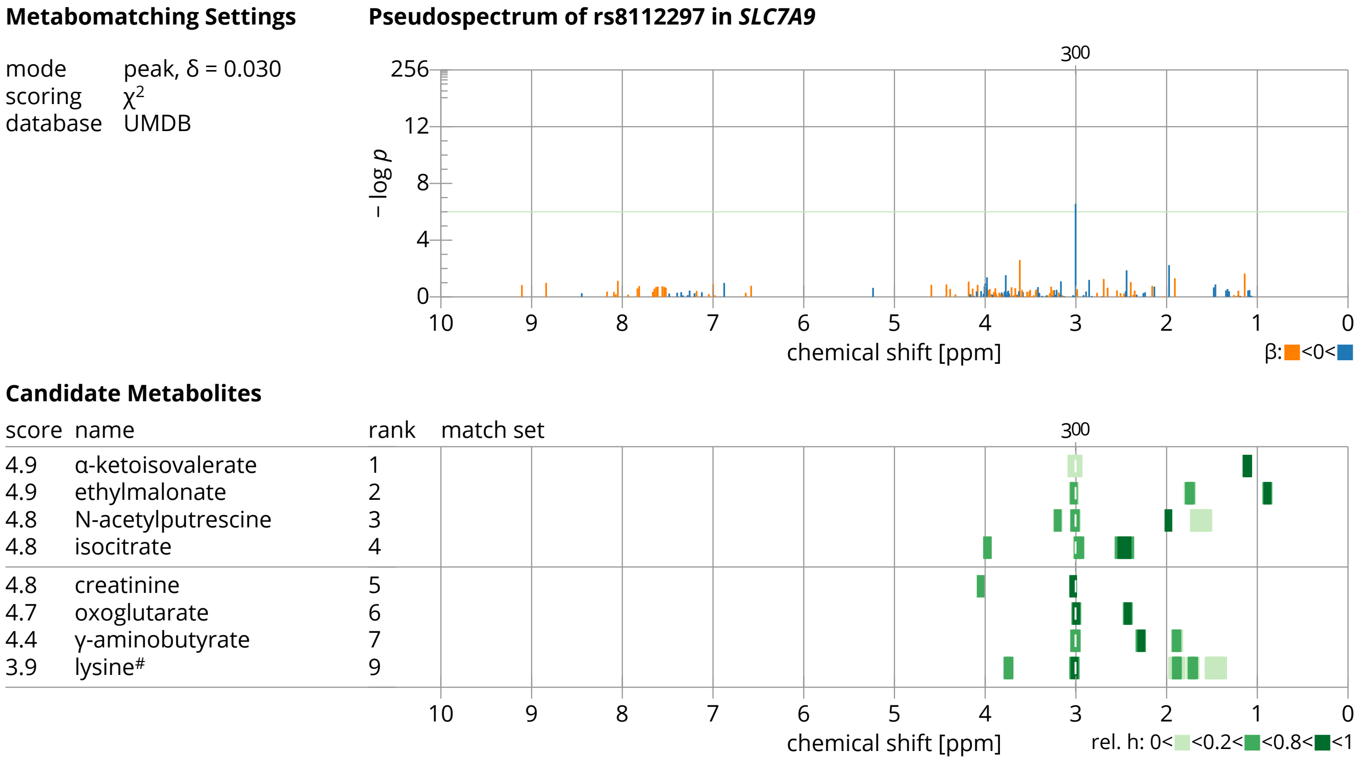

(V) SHIP, *SLC7A9*. Peak-mode, χ^2-scoring metabomatching. The rs811297 pseudospectrum is characterized by a single significantly associated feature: feature 3.00. The main peak of the spectrum of lysine matches feature 3.00, but the spectrum contains four additional, unmatched, peak clusters. Lysine is therefore outranked by other metabolites that match feature 3.00, but have fewer additional unmatched peak clusters. Compare to the stronger match of lysine with the *SLC7A9* SNP pseudospectrum in CoLaus (Fig S1I).

Navigation:

CoLaus	*SLC6A20*	*AGXT2*	*SOSTDC1*	*PYROXD2*	*SLC6A13*	*HPD*	*HPD/M*	*PNMT*	*SLC7A9*	*UPS9*
SHIP	*DAB1*	*CPS1*	*CPS1/2c*	*CPS1/M*	*CPS1/M/2c*	*XYLB*	*XYLB/M*	*SLC6A20*	*ENTPPL*	*SLC6A19*	*AGXT2*
	DMGDH	*SLC36A2*	*NAT2*	*NAT2/±*	*SLC6A13*	*HPD*	*HPD/2c*	*HPD/M*	*HPD/M/2c*	*PNMT*	*SLC7A9*
