# Supplementary material for: Metabomatching: Using genetic association to identify metabolites in proton NMR spectroscopy
Source: PLoS Comput Biol. 2017 Dec 1;13(12):e1005839. doi: 10.1371/journal.pcbi.1005839 (PMC5711027; doi:10.1371/journal.pcbi.1005839)
Supplement: S3 Fig — The pseudospectrum of rs10774021 in SLC6A13 is characterized by a single significantly associated feature: feature 1.19. (A) With χ2-scoring metabomatching, the association with feature 1.19 is sufficient for metabolites of spectrum matching feature 1.19, including the reference metabolite 3-aminoisobutyrate, to obtain top ranks. (B) With Z-scoring metabomatching, metabolites of spectrum matching 1.19 are outranked by metabolites matching peaks in the region between 3.6 and 3.9 ppm, which produces, on its own, a score of 11.8. (C) The 3.6 to 3.9 ppm region is characterized by strong correlation. By applying decorrelation, with λ = 0.5, the score produced by the region, on its own, is reduced to 0.8. Correspondingly, with decorrelation, 3-aminoisobutyrate outranks most metabolites that, without decorrelation, ranked highly by matching the 3.6 to 3.9 ppm region. (PDF) [file pcbi.1005839.s006.pdf]

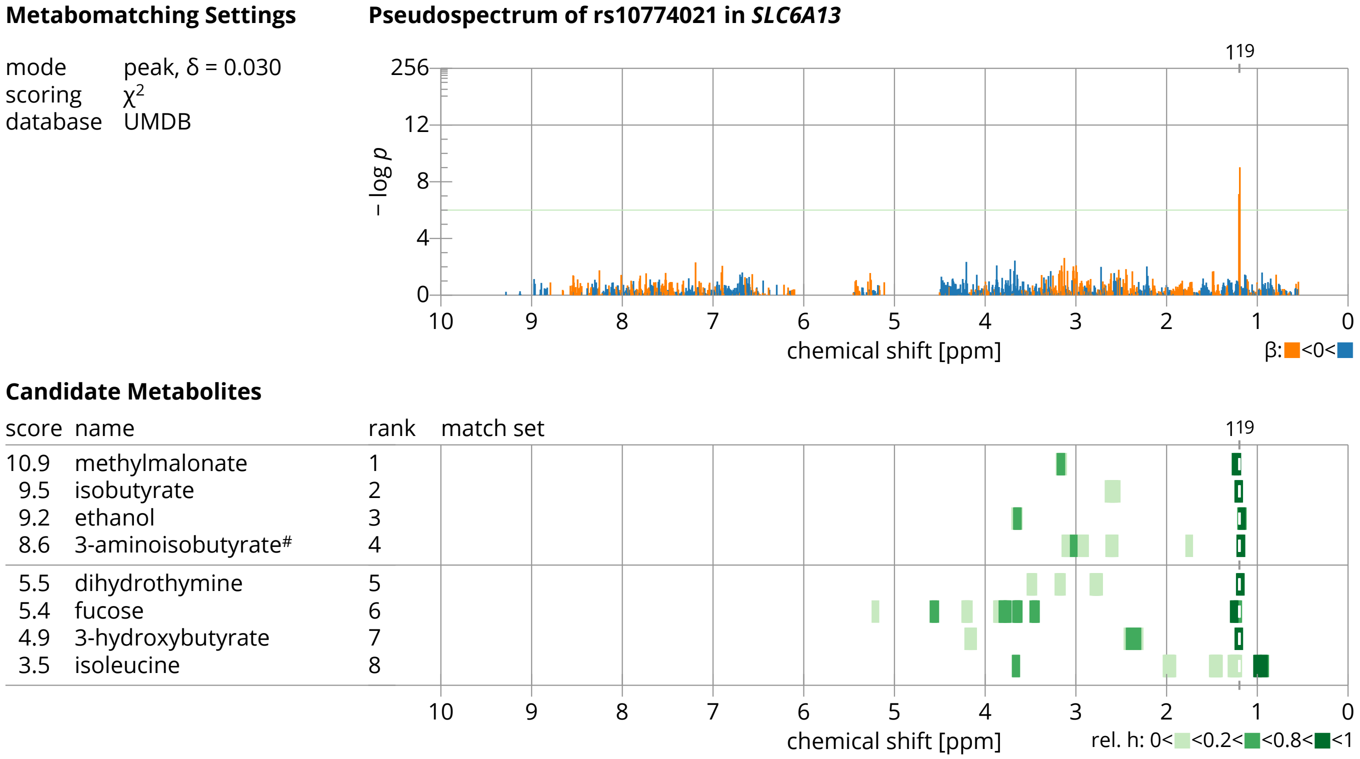

(A) χ^2-scoring metabomatching of *SLC6A13* pseudospectrum.

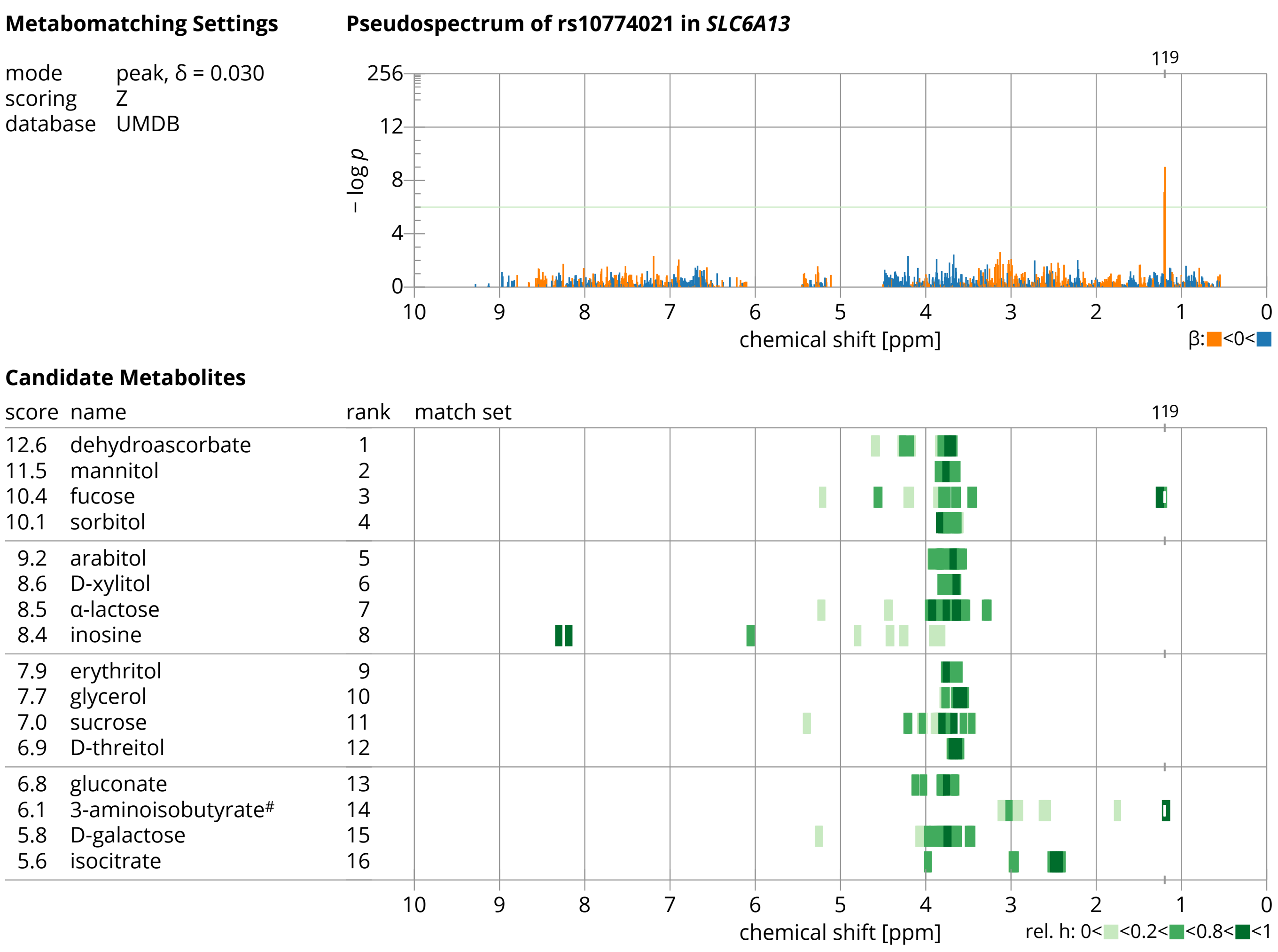

(B) Z-scoring metabomatching of *SLC6A13* pseudospectrum.

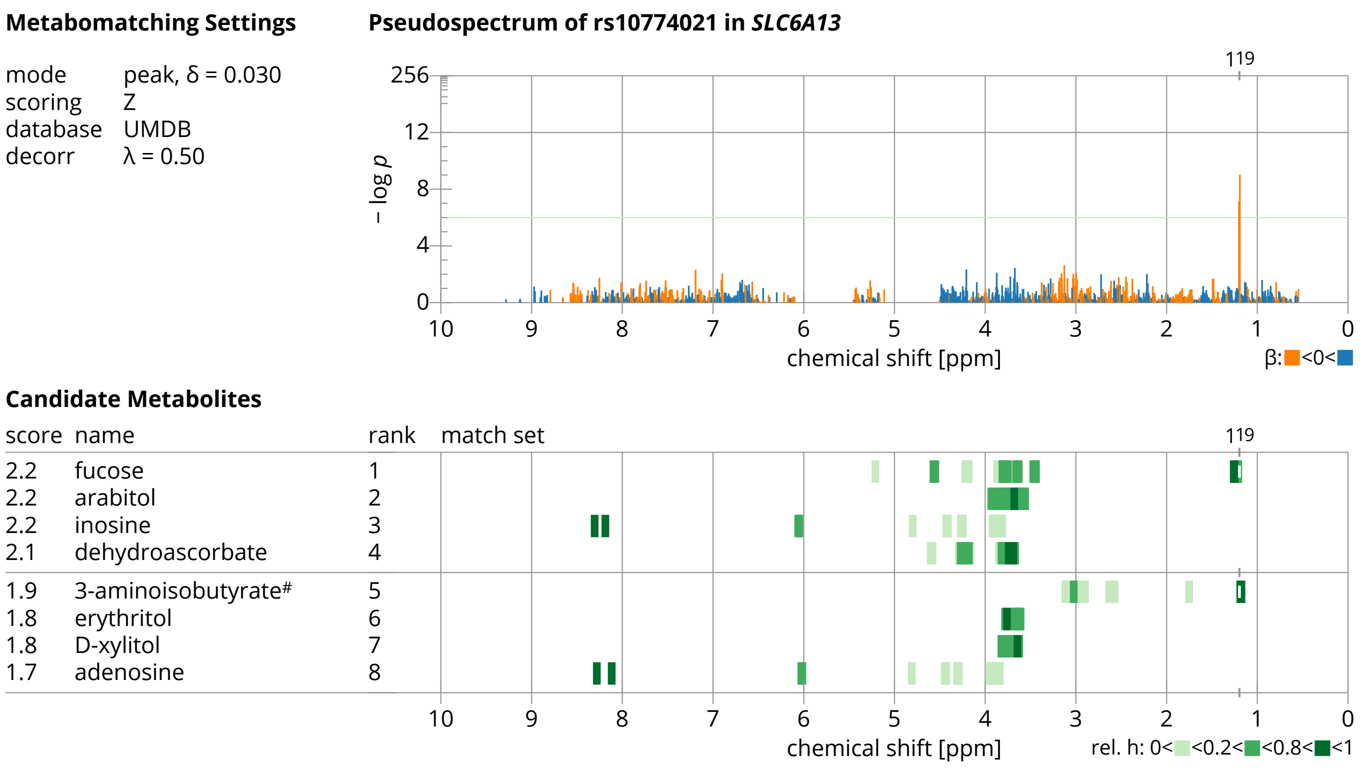

(C) Z-scoring metabomatching with decorrelation of *SLC6A13* pseudospectrum.
