# Supplementary figures and images for: Metabomatching: Using genetic association to identify metabolites in proton NMR spectroscopy
Source: PLoS Comput Biol. 2017 Dec 1;13(12):e1005839. doi: 10.1371/journal.pcbi.1005839 (PMC5711027; doi:10.1371/journal.pcbi.1005839)

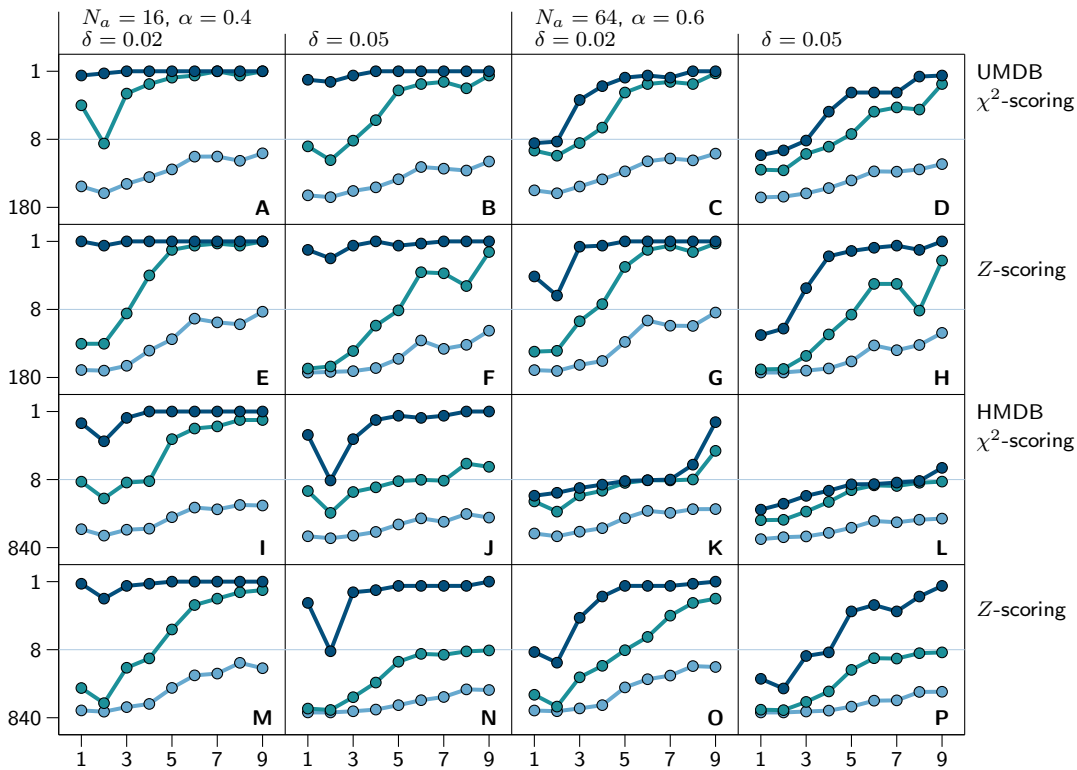

Supplement: S4 Fig — Median r90m, with metabolites grouped by their rounded size, and from light to dark blue, β = 0.2, 0.4, 1.6. We show results for UMDB and HMDB, neighborhood parameters δ = 0.02 and 0.05, χ2- and Z-scoring, and two genetic noise levels, defined by Na and α set to 16 and 0.4, and 64 and 0.6, respectively. From these cases, we see that the performance of metabomatching is consistently stronger in the smaller spectral database UMDB, and for smaller δ. For the weaker genetic noise, the median r90m is equal to, or close to, 1, for sufficiently large β, except in the case of δ = 0.05 in HMDB for sm = 2. For strong genetic noise, metabomatching performance is consistently poorer, with r90m often far from 1. χ2-scoring performs better than Z-scoring under weak genetic noise. When genetic noise is strong, however, Z-scoring performs almost invariably better: when β is large, there are sizes sm for which Z-scoring produces r90m close to 1 while χ2-scoring fails to do so. (PDF) [file pcbi.1005839.s007.pdf]
